# Bioactive Selenium Peptides Rescue Dopaminergic Neurodegeneration via Coordinated Signal Remodeling and Redox Reinforcement

**DOI:** 10.3390/cells15181628

**Published:** 2026-09-08

**Authors:** Xue Hou, Lin Luo, Ruojia Li, Yuying Li, Zhiyong Wang, Yangyang Wu

**Affiliations:** 1 Hubei Engineering Research Center of Selenium Food Nutrition and Health Intelligent Technology, Hubei Minzu University, Enshi 445000, China; 202330395@hbmzu.edu.cn (X.H.); 202430418@hbmzu.edu.cn (L.L.); 202530419@hbmzu.edu.cn (R.L.); 202530436@hbmzu.edu.cn (Y.L.); 2 College of Biological and Food Engineering, Hubei Minzu University, Enshi 445000, China; 3 School of Life Sciences, Westlake University, Hangzhou 310024, China

**Keywords:** selenium-modified bioactive peptide, Parkinson’s disease, selenium utilization disorder, redox homeostasis, *C. elegans*

## Abstract

**Highlights:**

(1) Cerebrospinal fluid proteomics identifies selective loss of glutathione peroxidase 3 in Parkinson’s disease, revealing a functional selenium utilization disorder rather than systemic deficiency.
(2) A selenium-modified peptide composite is fabricated from plant-derived peptides via controlled selenization, with organic-bound selenium that avoids the dose-dependent toxicity of inorganic salts.
(3) The selenopeptide material exhibits superior neuroprotective activity over inorganic selenium in a worm Parkinson’s model, demonstrating structure-dependent bioactivity enhancement.
(4) Multi-omics analysis links the material’s action to coordinated regulation of dephosphorylation, axon guidance, and endogenous antioxidant networks, establishing a materials-biology relationship.

**Abstract:**

Selenium-dependent antioxidant systems are critical for neuronal redox balance, but whether Parkinson’s disease (PD) involves systemic selenium deficiency or selective utilization impairment remains unclear. Using Parkinson’s Progression Markers Initiative (PPMI) proteomic data, we identified specific downregulation of glutathione peroxidase 3 (GPX3) in cerebrospinal fluid in PD, suggesting a compartment-specific alteration in GPX3-related antioxidant defense rather than a uniform systemic selenium deficit. Inorganic selenium sources suffer from low bioavailability and narrow therapeutic windows. We therefore developed a selenium-enriched peptide fraction (IPP-Se-F12) via controlled selenization of *Idesia polycarpa* Maxim. cake meal peptides, and characterized its selenium content, size distribution, and radical-scavenging activity. In SH-SY5Y and nematode models, IPP-Se-F12 attenuated 6-hydroxydopamine (6-OHDA)-induced cell death and rescued locomotor and dopamine-dependent behaviors more effectively than sodium selenite treatment. RNAseq further showed that IPP-Se-F12 treatment was associated with transcriptional changes in dephosphorylation, axon-guidance, and GPX3-centered antioxidant networks, including increased expression of *gpx-3* and *gpx-5*. This study supports IPP-Se-F12 as a plant-derived selenium-associated peptide fraction with protective activity in cellular and nematode models and identifies GPX-related antioxidant regulation as a candidate mechanism for further investigation.

## 1. Introduction

Parkinson’s disease (PD) is the second most prevalent neurodegenerative disorder, characterized pathologically by the selective degeneration of dopaminergic (DA) neurons in the substantia nigra and the aggregation of Lewy bodies [1,2]. Cumulative evidence from clinical trials and experiments supports oxidative stress as a core driver of this neurodegenerative process, stemming from mitochondrial dysfunction, the accumulation of reactive oxygen species (ROS), and an impaired redox buffering capacity [3,4]. DA neurons, in particular, are uniquely sensitive to oxidative damage due to their high metabolic demands, dopamine auto-oxidation, and limited antioxidant reserves [5]. The vulnerability renders them prone to preferential loss during redox imbalance, marking them as a critical pathogenic node in PD.

Selenium (Se) is an essential trace element that plays a pivotal role in maintaining redox homeostasis via its incorporation into 25 selenoproteins, including glutathione peroxidases, thioredoxin reductases, and other redox-active enzymes [6,7]. These enzymes, especially in the central nervous system (CNS) are pivotal in maintaining mitochondrial homeostasis, mitigating lipid peroxidation, and modulating apoptotic signaling [8,9]. Despite its known importance, the clinical relationship between selenium and PD remains controversial. Clinical studies have reported heterogeneous changes in circulating selenium levels in PD patients, and conventional selenium supplementation therapies have yielded unreliable efficacy [10,11,12]. This raises the possibility that altered utilization of particular selenoproteins, rather than a uniform systemic deficiency, may be relevant to PD.

Notably, the brain exhibits a hierarchical priority for selenium retention [13]. Even under conditions of systemic deficiency, cerebral selenium levels and selenoprotein expression are preferentially maintained via the blood–brain barrier’s SELENOP-ApoER2 transport axis [13,14]. This prioritization suggests that neurodegeneration-related selenium dysregulation cannot be explained solely by insufficient dietary intake or impaired systemic transport. Through deep mining of the Parkinson’s Progression Markers Initiative (PPMI) proteomic datasets, we observed reduced glutathione peroxidase 3 (GPX3) abundance in cerebrospinal fluid (CSF), whereas SELENOP and TXNRD1 were not significantly altered, suggesting that patients with PD may exhibit functional selenium utilization disorder rather than a global deficiency. Consistently, accumulating evidence points toward the selective vulnerability of specific selenoproteins within the CNS, where the dysfunction of specific Se-dependent enzymes may exert disproportionate pathological effects [9]. GPX3 is a secreted glutathione peroxidase expressed by several cell types, and reduced GPX3 has been associated with oxidative and inflammatory injury in other systems [15,16]. Our results suggest that the pathogenesis of PD involves a state of functional selenium utilization disorder, leading to compromised antioxidant capacity. Therefore, intervention strategies should focus on restoring functional selenium utilization to reestablish antioxidant efficacy, rather than indiscriminately increasing total selenium intake.

Previous investigations into selenium-based therapeutics have largely centered on inorganic salts, organic compounds, or selenium-enhanced nanomaterials, primarily aimed at augmenting bioavailability to mitigate oxidative stress [17]. Compared with inorganic selenium, organic selenium is taken up more rapidly by the liver. Various excretion indices, including fecal, urinary, and total excretion, indicate that organic selenium exhibits lower excretion rates than sodium selenite (Na_2_SeO_3_), resulting in longer retention times in vivo, with absorption efficiency of dietary selenium reaching approximately 90% [18]. Although organic selenium can be more effectively absorbed and utilized, it also raises concerns regarding excessive tissue accumulation and associated toxicity when organic selenium is supplemented in an uncontrolled or long-term high-dose manner, such as an increased risk of type 2 diabetes [19]. Similarly, selenium nanomaterials, while advantageous in their ability to cross the blood–brain barrier and deliver selenium to the brain, suffer from inherent limitations such as instability, aggregation, and poor recyclability [20]. Excessive intake may also lead to the accumulation of surplus selenium-containing amino acids, ultimately resulting in protein dysfunction.

Bioactive peptides are attractive scaffolds for selenium-containing formulations because their composition and processing can be tuned and because many food-derived peptides possess intrinsic antioxidant activity [21,22]. Compared to inorganic selenium compounds, organic selenium forms, particularly selenium-enriched peptides, offer enhanced bioavailability and lower toxicity profiles [23]. The antioxidant activity of selenium-enriched peptides may reflect the combined contributions of its components: selenium serves as the catalytic redox center essential for selenoprotein activity, while peptide sequence characteristics (such as amino acid composition, hydrophobicity, terminal residues, and chain length), modulate free radical scavenging capacity and cellular interactions [21]. Extensive research using plant-derived selenium-enriched peptides has demonstrated efficient antioxidant effects in chemical, cellular, and animal models, supporting their potential as functional biomaterials for redox regulation [24]. Despite these advances, several limitations hinder the translation of selenium-enriched peptides into interventions for neurodegenerative diseases. Existing studies mostly focus on peripheral oxidative stress models, aging, or liver injury, with limited exploration regarding the background of dopaminergic neurodegeneration and protective mechanisms [3]. Furthermore, naturally extracted selenium-enriched peptides are often obtained at low yields and are compositionally heterogeneous, making a rigorous distinction between molecular composition, colloidal behavior, and biological activity essential.

*Idesia polycarpa* Maxim. cake meal is a protein-rich by-product of oil processing with limited direct application in food and feed. Previous studies have demonstrated its antioxidant potential and identified antioxidant peptides derived from its protein hydrolysates. Therefore, it was selected as a plant-derived peptide source for selenium modification. In this study, we prepared an *Idesia polycarpa* Maxim. cake meal peptide-selenium formulation (IPP-Se) and used response surface methodology to determine how processing variables influenced selenium loading. We characterized the formulation by spectroscopy, particle-size analysis, microscopy, amino-acid analysis, and LC-MS/MS to evaluate its structural properties and antioxidant activity. Using a 6-hydroxydopamine (6-OHDA) induced *Caenorhabditis elegans* model and SH-SY5Y cell models, we evaluated the protective effects of IPP-Se-F12 against dopaminergic dysfunction and cellular injury. Furthermore, RNA-seq analysis suggested that IPP-Se-F12 treatment was associated with transcriptional responses involving protein dephosphorylation, axon-guidance-related pathways, and GPX3-related antioxidant defense [25].

## 2. Method

### 2.1. PPMI Cohort Proteomic Analysis

Large-scale, unbiased proteomic datasets were obtained from PPMI database. Proteomic data used in this study were obtained from the Parkinson’s Progression Markers Initiative (PPMI) database on 29 July 2024 (www.ppmi-info.org/access-data-specimens/download-data). Specifically, Project 177 used the DIA-MS platform to analyze CSF samples, whereas Project 222 used the Olink Explore 384 Neurology panel to analyze plasma samples. Olink protein expression data were provided as Normalized Protein Expression (NPX) values. The analysis included CSF and plasma samples from normal controls (Control), prodromal individuals (Prodromal), and patients with PD. Key proteins associated with selenium-dependent redox homeostasis were specifically selected and quantitatively analyzed, including glutathione peroxidase 3 (GPX3), selenoprotein P (SELENOP), and thioredoxin reductase 1 (TXNRD1).

For statistical analysis and biomarker evaluation, expression levels of selenium-related proteins were standardized using Z-score normalization, followed by between-group comparisons. Univariate logistic regression analysis was applied to assess the association between selenium-related protein levels and PD risk. Receiver operating characteristic (ROC) curves and the area under the curve (AUC) were used to evaluate the discriminatory power of individual proteins for PD status. Pearson correlation analysis was performed to examine the relationships between CSF selenium-related proteins and biomarkers associated with lysosomal function (PSAP), lipid metabolism (LCAT), mitochondrial apoptosis (CYCS), and proteostasis (APP).

### 2.2. Optimization of Selenium Peptide (IPP-Se) Preparation

#### 2.2.1. Extraction and Enzymatic Hydrolysis of *Idesia polycarpa* Maxim. Cake Meal Proteins

The extraction of *Idesia polycarpa* Maxim. cake meal protein was performed according to the previous study. Briefly, the raw materials were first dried, pulverized, and defatted to obtain a defatted powder. Protein extraction was carried out using 1 M NaOH adjusted to pH 10 at 40 °C for 2 h. Following extraction, the mixture was centrifuged to collect the supernatant. The pH of the supernatant was adjusted to 4 using 1 M HCl, and the solution was allowed to stand for 1 h. The resulting precipitate was collected by centrifugation and subsequently freeze-dried.

*Idesia polycarpa* Maxim. cake meal protein hydrolysates were prepared using a complex protease under the following conditions: the protein substrate concentration was set at 2% (*w*/*v*) with an enzyme-to-substrate ratio of 3% (*w*/*w*). The pH was adjusted to 8 using 1 M NaOH and 1 M HCl, and the hydrolysis reaction was conducted at 46 °C for 2.7 h. Subsequently, the mixture was incubated in a 90 °C water bath for 10 min (to terminate the enzymatic reaction), cooled to room temperature, and centrifuged at 10,000× *g* for 20 min. The resulting supernatant was collected and lyophilized for further analysis.

#### 2.2.2. Preparation of *Idesia polycarpa* Maxim. Cake Meal Peptides-Selenium (IPP-Se) Chelate

Prior to the formal optimization, the effects of three controllable variables, which are reaction temperature (50, 60, 70, 80, and 90 °C), pH (7.0, 8.0, 9.0, 10.0, and 11.0), and the volume ratio of 0.5 M sodium selenite (Na_2_SeO_3_) solution to 3% (*w*/*v*) peptide solution (3:1, 2:1, 1:1, 1:2, and 1:3), on the preparation of IPP-Se were investigated. The mixture was allowed to chelate for 60 min and then cooled under ambient conditions to room temperature (approximately 25 °C). After multiple rounds of centrifugation, the supernatant was collected and mixed with five volumes of 95% ethanol (*v*/*v*). The mixture was sealed with plastic wrap and kept at 4 °C for 12 h to facilitate precipitation. Subsequently, the solution was centrifuged at 6000 rpm for 10 min, and the resulting precipitate was washed several times with 75% ethanol. The final precipitate was collected and lyophilized to obtain IPP-Se. The optimal preparation conditions for IPP-Se were further determined and refined using a Box–Behnken design (BBD) under response surface methodology (RSM).

### 2.3. Determination of Se Content

The total selenium content was determined using atomic fluorescence spectrometry (AFS) with an atomic fluorescence spectrophotometer (BAF2000; Beijing Baode Instrument Co., Ltd., Beijing, China) in accordance with the Chinese National Food Safety Standard GB 5009.93-2017 [26]. Briefly, samples were weighed into digestion tubes, mixed with 10 mL of concentrated HNO_3_ (65–68%, *w*/*w*) and 2 mL of 30% (*w*/*w*) H_2_O_2_, and agitated thoroughly. The mixture was then digested using a microwave digestion system (Touchwin 2.0; APC Science and Technology Co., Ltd., Chengdu, China). After cooling, 5 mL of 6 M HCl was added to the digestate. The mixture was heated in a graphite acid removal system (GS40; APC Science and Technology Co., Ltd., Chengdu, China) until the solution became clear and colorless with the appearance of white smoke, ensuring the reduction in hexavalent selenium (Se~(6+)) to tetravalent selenite (Se~(4+)). Once cooled, the solution was diluted to a final volume of 10 mL with HCl. A blank sample, consisting of 10 mL HNO_3_ and 2 mL H_2_O_2_ without the specimen, was prepared and processed simultaneously following the same digestion and treatment procedure.

$$X=\left(\rho -\rho_{0}\right)\times \frac{V}{m}/1000$$

In the formula,

*X*: The selenium content in the sample, expressed in milligrams per kilogram (mg/kg) or milligrams per liter (mg/L);

*ρ*: The mass concentration of selenium in the sample solution, expressed in micrograms per liter (μg/L);

*ρ*_0_: The mass concentration of selenium in the blank solution, expressed in micrograms per liter (μg/L);

*V*: The total volume of the digested sample solution, expressed in milliliters (mL);

*m*: The weight or volume of the sample, expressed in grams (g) or milliliters (mL);

1000: A unit-conversion factor accounting for the use of V in mL when ρ and ρ_0_ are expressed in μg/L; the resulting μg/g value is numerically equivalent to mg/kg.

### 2.4. Characterization of IPP and IPP-Se

#### 2.4.1. Spectral Analysis

The samples were mixed with potassium bromide (KBr) and pressed into translucent pellets. Fourier-transform infrared (FT-IR) spectra were recorded using an FT-IR spectrometer (Nicolet iS5; Thermo Electron Scientific Instruments LLC, Madison, WI, USA) across a wavenumber range of 4000 to 500 cm^−1^. For secondary-structure estimation, the amide I region was smoothed, evaluated by second-derivative-assisted peak identification, and curve-fitted using PeakFit (v4.12) and relative peak areas were assigned to alpha-helix, beta-sheet, beta-turn, and random-coil components.

For UV-Vis analysis, samples were prepared in deionized water at a concentration of 0.01 mg/mL. The absorbance was measured using a UV-Vis spectrophotometer (UV-9000S; Shanghai Yuanxi Instrument Co., Ltd., Shanghai, China) within a wavelength range of 200 to 600 nm.

#### 2.4.2. Particle Size Analysis

The particle size of the samples was measured and analyzed using a Zetasizer (Zetasizer Nano ZS90; Malvern Instruments Ltd., Malvern, UK). The specific experimental parameters were as follows: deionized water was used as the dispersive medium; the equilibration time was set to 120 s; the analysis was conducted at a temperature of 25 °C, and the sample concentration was maintained at 1 mg/mL.

#### 2.4.3. Microscopic Morphology Analysis

The microscopic morphology and elemental composition of the samples were analyzed using a field-emission scanning electron microscope (GeminiSEM 300; Carl Zeiss Microscopy GmbH, Oberkochen, Germany). Briefly, the dried samples were mounted onto conductive adhesive tape, subjected to vacuum treatment, and sputter-coated with a thin layer of gold to enhance conductivity. The surface morphology was then observed and recorded at various magnifications.

### 2.5. Amino Acid Composition Analysis

The amino acid compositions of IPP and IPP-Se were determined using an automated amino acid analyzer (Biochrom 30+; Biochrom Ltd., Cambridge, UK) in accordance with the Chinese National Food Safety Standard GB 5009.124-2016 [27].

### 2.6. Analysis of Antioxidant Activity

The ABTS radical scavenging activity was determined according to the method described by Dou et al. [28] with modifications. Briefly, equal volumes of 7 mM ABTS solution and 2.45 mM K_2_S_2_O_8_ were mixed and incubated in the dark at 4 °C for 16 h to generate the ABTS radical cation. Before use, the ABTS stock solution was diluted with ultrapure water to reach an absorbance of 0.7 ± 0.02 at 734 nm. Subsequently, 0.2 mL of the protein sample solution at various concentrations was added to 4 mL of the diluted ABTS solution. The mixture was allowed to react for 6 min in the dark, and the absorbance was measured at 734 nm. The ABTS radical scavenging activity was calculated using the following formula:

$${\mathrm{ABTS}}^{+}\;\mathrm{scavenging}\;\mathrm{rate}\;(\%)\;=\;[1\;-\;(A_{1}\;-\;A_{2}\;)/A_{0}]\;\times \;100$$

where

*A*_1_: Absorbance of the mixture containing the sample solution and the diluted ABTS solution;

*A*_2_: Absorbance of the mixture containing the sample solution and ultrapure water;

*A*_0_: Absorbance of the mixture containing ultrapure water and the diluted ABTS solution.

Hydroxyl radical(-OH)scavenging ability was determined on the basis of the method of Li et al. [29], with slight modification. Briefly, different concentrations of sample solution, 9 mM salicylic acid (ethanol solution), and 9 mM FeSO_4_ solution were evenly mixed at a ratio of 1:1:1, and then one volume of 9 mM H_2_O_2_ solution was added to start reaction. The absorbance was measured at 510 nm after incubation at 37 °C for 30 min. The -OH scavenging rate was calculated as follows:

$$\mathrm{OH}\;\mathrm{scavenging}\;\mathrm{rate}\;(\%)\;=\;[1\;-\;(A_{1}\;-\;A_{2}\;)/A_{0}]\;\times \;100$$

where

*A*_1_: Absorbance of the mixture containing the sample solution and the H_2_O_2_ solution;

*A*_2_: Absorbance of the mixture containing the sample solution and ultrapure water;

*A*_0_: Absorbance of the mixture containing ultrapure water and the H_2_O_2_ solution.

### 2.7. Separation and Identification of Idesia polycarpa Maxim. Cake Meal Peptide-Selenium Complex

#### 2.7.1. Ultrafiltration

IPP-Se was dissolved in ultrapure water to prepare a 10 mg/mL solution, which was then passed through a 0.45 μm membrane filter. The filtrate was transferred into a 3 kDa molecular weight cut-off (MWCO) ultrafiltration tube for equilibration. Centrifugation was performed at 4000 r/min for 30 min. The retentate (upper fraction, >3 kDa) and the permeate (lower fraction, <3 kDa) were collected separately. The permeate (<3 kDa) and retentate (>3 kDa) were designated F1 and F2, respectively. The selenium content of each fraction was determined, and the fraction exhibiting higher content was collected for further separation and purification.

#### 2.7.2. Dextran Gel Column Chromatography Separation

Sephadex G-25 gel was washed and allowed to swell for 48 h before being packed into a glass column (1.6 cm × 80 cm) and equilibrated. The IPP-Se fraction previously identified with the highest selenium content was prepared as a 50 mg/mL solution. A 2 mL aliquot of this solution was loaded onto the column and eluted with ultrapure water as the mobile phase at a flow rate of 0.6 mL/min. The eluent was monitored at 214 and 280 nm, and fractions corresponding to different chromatographic peaks were collected at specific time intervals. The components corresponding to the same peak area were concentrated and lyophilized. The wavelength of 214 nm was selected because peptide bonds exhibit strong UV absorption in this region, allowing sensitive and general detection of peptide-containing fractions. In contrast, absorbance at 280 nm primarily reflects peptides containing aromatic amino acid residues, particularly tryptophan and tyrosine. Therefore, simultaneous monitoring at 214 and 280 nm provided complementary information on the overall peptide elution profile and the presence of aromatic amino acid-containing peptide fractions.

#### 2.7.3. Mass Spectrometry Identification

The selenium-associated peptide fraction obtained by gel filtration was analyzed by high-performance liquid chromatography coupled with tandem mass spectrometry (LC-MS/MS). The samples were first desalted using C18 solid-phase extraction (SPE) cartridges and then separated using an EASY-nLC 1200 nano-flow liquid chromatography system (Thermo Fisher Scientific, Waltham, MA, USA) equipped with a reversed-phase LC column (0.15 mm × 150 mm, RP-C18; Column Technology Inc., Lombard, IL, USA). The mobile phases consisted of solution A (0.1% formic acid in water) and solution B (0.1% formic acid in 84% acetonitrile). The LC column was equilibrated with 95% solution A. Samples were loaded onto Zorbax 300SB-C18 peptide traps (Agilent Technologies, Wilmington, DE, USA) and separated using a gradient elution profile. Mass spectrometry analysis was performed on a Q Exactive HF-X (Thermo Fisher Scientific, Bremen, Germany) mass spectrometer. The resulting MS/MS data were processed using Proteome Discoverer software and searched against UniProtKB protein entries assigned to the taxonomic group Flacourtieae. Because a comprehensive species-specific proteome database for *Idesia polycarpa* is not currently available, some peptide-spectrum matches may correspond to homologous proteins from closely related taxa within this group. Therefore, these matches were used primarily for peptide-sequence identification and should not be interpreted as definitive assignments of the parent proteins in *Idesia polycarpa*.

#### 2.7.4. In Silico Peptide Screening and Molecular Docking

From the LC–MS/MS assignments, nine peptides with a PeptideRanker score > 0.5 and a hydrophobic amino acid proportion > 50% were selected for molecular docking. The PeptideRanker score was used as an in silico indicator of potential bioactivity, whereas the hydrophobic amino acid criterion was applied because hydrophobic residues are frequently associated with the radical-scavenging activity of food-derived peptides. The observed peptide lengths of 10–16 amino acids were not used as a selection criterion. The crystal structure of the human KEAP1 Kelch domain (PDB ID: 5WFV) was obtained from the RCSB Protein Data Bank. The Kelch domain was selected as an exploratory receptor because it is a well-characterized peptide-recognition domain that binds the NRF2 ETGE and DLG motifs in the KEAP1–NRF2 antioxidant pathway, while the candidate peptides were not selected based on sequence similarity to these motifs. Three-dimensional structures of the candidate peptides were independently constructed using Chem3D (v14.00.117) and used as the starting conformations for molecular docking. The α-helix, β-sheet, β-turn, and random-coil proportions determined by FTIR for the bulk IPP and IPP-Se preparations were not imposed as structural constraints on the individual peptides because these values represent ensemble-averaged structural characteristics of the heterogeneous peptide preparations rather than experimentally determined conformations of the individual LC–MS/MS-identified peptides. Molecular docking between the candidate peptides and the KEAP1 Kelch domain was performed using AutoDock Vina (v1.5.7). The resulting docking poses were evaluated based on their predicted binding energies and interacting amino acid residues, and representative peptide–KEAP1 complexes were visualized using PyMOL (v3.1.6.1). Docking was used to generate a testable antioxidant-signaling hypothesis and was not interpreted as evidence of cellular target engagement or pathway activation.

### 2.8. Maintenance and Culture of C. elegans

All *C. elegans* strains were maintained at 20 °C on Nematode Growth Medium (NGM) plates seeded with *E. coli* OP50. For all experiments, worms were synchronized to the L4 larval stage. Synchronized eggs were obtained using the bleaching method (lysis solution containing 10% NaClO and 2 M NaOH), followed by multiple washes with M9 buffer to remove residual debris. To achieve age synchronization, the harvested eggs were incubated in M9 buffer at 20 °C for 12 h until they reached the L1 stage via starvation-induced arrest. The synchronized L1 larvae were then transferred to fresh NGM plates and cultured at 20 °C for an additional 48 h to reach the L4 stage. To prevent progeny interference in subsequent assays, 5-fluoro-2′-deoxyuridine (FUDR) was added to the media at a final concentration of 150 μM for liquid NGM and 80 μM for solid NGM, unless otherwise specified. All experiments were performed in triplicate as independent biological replicates.

### 2.9. Lifespan Assay

The lifespan assay was conducted using N2 (wild-type) worms treated with IPP, IPP-Se, purified IPP-Se, and N-acetylcysteine (NAC, 100 μg/mL) as a positive control. When the NGM cooled to approximately 50–55 °C, it was supplemented with the various samples and mixed with *E. coli* OP50 to achieve a final sample concentration of 20 μg/mL (nominal medium concentration). For each experimental group, 100 synchronized worms were observed. The number of surviving worms was recorded every 48 h, at which time the worms were transferred to fresh plates. This process continued until all worms had died. Additionally, because IPP, IPP-Se, and IPP-Se-F12 are heterogeneous peptide preparations without a single defined molecular weight, their concentrations cannot be reliably expressed on a molar basis. Therefore, all treatments were administered and compared using mass concentrations (μg/mL) to maintain a consistent dosing basis across groups.

### 2.10. PD Model in C. elegans

6-OHDA-induced *C. elegans* PD model was used to evaluate the prophylactic potential of different selenium formulations on dopaminergic neurons. Synchronized pre-conditioned L4-stage wild-type N2 nematodes were exposed to 10 mM 6-OHDA in M9 buffer for 1 h to induce dopaminergic neuronal damage [30]. Experimental groups included a solvent control group, a 6-OHDA injury group, and various intervention groups, including crude peptide, crude selenium peptide, purified selenium peptide, sodium selenite, and the positive control NAC (100 μg/mL). All compounds were added to NGM plates at a final concentration of 20 μg/mL.

### 2.11. Behavioral Assays

Pharyngeal pumping frequency within a 20 s interval was recorded to assess feeding ability. Locomotor function was evaluated by counting body bends within 20 s. Dopaminergic neuronal integrity was assessed using the basal slowing response (BSR) assay, calculated as: BSR = (locomotion rate off food − locomotion rate on food)/locomotion rate on food. All results are presented as mean ± standard deviation (SEM). Statistical significance among groups was evaluated using one-way ANOVA.

### 2.12. SH-SY5Y Neurotoxicity Assay

SH-SY5Y human neuroblastoma cells (kindly gifted by Professor Shanhui Liu from the Second Hospital of Lanzhou University (Lanzhou, China) were cultured in Dulbecco’s modified Eagle medium (DMEM) supplemented with 10% fetal bovine serum (FBS), 100 units/mL penicillin, and 100 μg/mL streptomycin in a humidified incubator with 5% CO_2_ at 37 °C. Purified IPP-Se was dissolved in sterile deionized water to prepare stock solutions, which were then diluted to final concentrations of 0.2 and 1 μM in culture medium (the pH of the stock solution was approximately 7.2–7.4 and was not adjusted). 6-OHDA was prepared as a 100 mM stock solution in sterile water containing 0.1% L-ascorbic acid under aseptic conditions. For the neuroprotection assay, cells were pre-incubated with IPP-Se at the indicated concentrations for 12 h. The medium was then removed, and cells were washed thoroughly with PBS to eliminate residual peptide. Subsequently, cells were challenged with 200 μM 6-OHDA for an additional 12 h. Following treatment, cell death was assessed by propidium iodide (PI, Beyotime Biotechnology, Shanghai, China) staining, and the percentage of PI-positive cells was quantified relative to the total cell population by manual counting under a fluorescence microscope (Axio Vert.A1, Carl Zeiss, Jena, Germany), with at least three random fields counted per well.

### 2.13. C. elegans RNA-Seq Analysis and Mechanistic Investigation

Synchronized L1 larvae were cultured under different experimental conditions until the L4 stage. Worms were incubated with 10 mM 6-OHDA and 200 mM ascorbic acid at 20 °C with shaking at 500 rpm for 1 h, followed by dilution with M9 buffer and recovery on NGM plates for 2 h. A sufficient number of worms was pooled for each biological replicate to obtain 1–2 μg of total RNA. Total RNA was extracted using TRIzol reagent, followed by DNase treatment and purification. RNA integrity was assessed using an Agilent Bioanalyzer, with samples exhibiting RNA integrity numbers (RIN) > 7.0 used for subsequent analysis.

Strand-specific RNA-seq libraries were constructed using systems such as SMARTer Apollo, starting with 1–2 μg of total RNA. After fragmentation, cDNA synthesis, adaptor ligation, and 12–15 cycles of PCR amplification were performed. Qualified libraries were sequenced on an Illumina NovaSeq platform with 150 bp paired-end reads, generating at least 10–20 million reads per sample, with a minimum of three biological replicates per group. Raw sequencing data were subjected to quality control using FastQC (v0.12.1), adaptor trimming with Cutadapt (v4.9), and alignment to the *C. elegans* reference genome (WBcel235) using HISAT2 (v2.2.1). Gene expression matrices were generated using featureCounts. Differentially expressed genes (DEGs) were identified using DESeq2 with thresholds of |log2 fold change| > 1 and false discovery rate (FDR) < 0.05. Gene Ontology (GO) and Reactome pathway enrichment analyses were subsequently performed.

Principal component analysis (PCA) was first conducted to evaluate global differences in expression profiles among experimental groups. For differentially expressed proteins, GO enrichment analysis was applied to assess their distribution across biological processes (BP), cellular components (CC), and molecular functions (MF), with particular emphasis on protein dephosphorylation, immune response, and synaptic transport. Reactome pathway analysis was used to identify regulated metabolic and signaling pathways, including axon guidance, semaphorin signaling, and neurodevelopment-related pathways. Core proteins associated with antioxidant defense and mitochondrial homeostasis (such as members of the SKN-1/Nrf2 and DAF-16/FOXO pathways, *gpx-3*/*4*/*5*/*8* GPX3) were further analyzed. Heatmaps and bar blots were generated to visualize expression changes in key stress-response genes (e.g., *sod-1*, *gpx-1*, *gst-4*) under different intervention conditions.

## 3. Results

### 3.1. Selenium Homeostasis Imbalance in Parkinson’s Disease Patients

**Figure 1:**
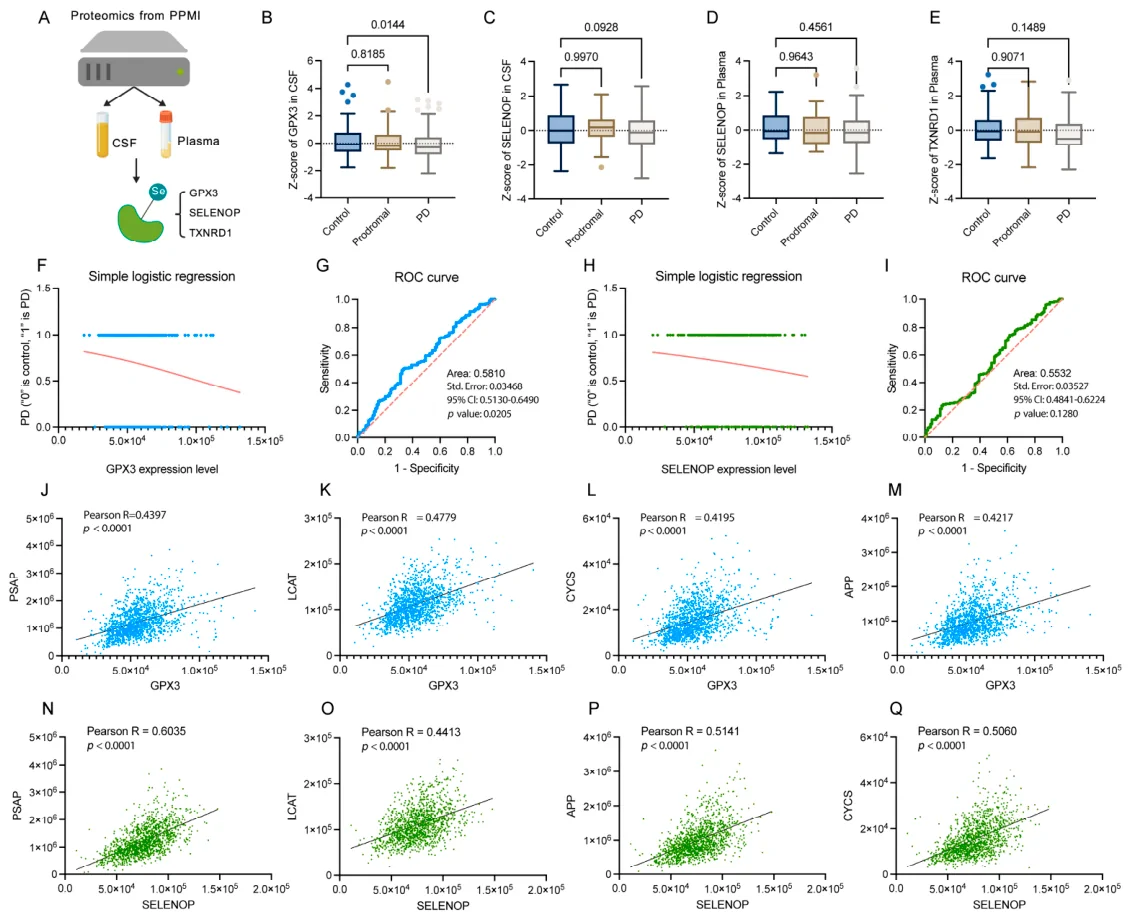
PPMI proteomics reveals an imbalance in selenium-dependent antioxidant functions in Parkinson’s disease and their association with key pathological pathways. (**A**) Schematic diagram of the proteomic analysis workflow for CSF and plasma samples from the PPMI cohort. Based on large-scale unbiased proteomic data, three proteins closely related to selenium-dependent redox homeostasis were screened and analyzed, including GPX3, SELENOP, and TXNRD1. (**B**) Comparison of CSF GPX3 expression levels (Z-scores) among the Control, Prodromal, and PD groups. Data are presented as Tukey-style box plots, where the box represents the interquartile range (IQR) with the median line, and whiskers extend to the most extreme points within 1.5 × IQR. Individual points beyond the whiskers represent outliers (values beyond 1.5 × IQR). The varying number of outliers reflects the inherent data variability rather than data exclusion. Statistical significance was determined by one-way ANOVA followed by Tukey’s post hoc test (*p* = 0.0144). (**C**–**E**) The same Tukey-style box plot format applies to CSF SELENOP (**C**), plasma SELENOP (**D**), and plasma TXNRD1 (**E**), with outliers plotted as individual points, while the dotted lines indicate the zero level. (**F**) Univariate logistic regression analysis based on CSF GPX3 expression levels to evaluate its ability to distinguish between PD and Control states. Reduced GPX3 expression is associated with an increased risk of PD. Blue dots represent the observed data, and the red line represents the fitted logistic regression model. (**G**) ROC curve analysis of CSF GPX3. GPX3 showed limited but statistically significant discriminatory ability for PD (AUC = 0.5810, 95% CI: 0.5130–0.6490, *p* = 0.0205). The blue line represents the ROC curve, and the red diagonal line represents random classification (AUC = 0.5). (**H**) Univariate logistic regression analysis based on CSF SELENOP expression levels. SELENOP expression showed weak predictive ability for PD status. Green dots represent the observed data, and the red line represents the fitted logistic regression model. (**I**) ROC curve analysis of CSF SELENOP. The discriminatory ability of SELENOP for PD was limited and did not reach statistical significance (AUC = 0.5532, *p* = 0.1280). The green line represents the ROC curve, and the red diagonal line represents random classification (AUC = 0.5). (**J**–**M**) Correlation analysis between CSF GPX3 and various PD-related proteins, including: PSAP (prosaposin, lysosomal function) (**J**), LCAT (lecithin–cholesterol acyltransferase, lipid metabolism) (**K**), CYCS (cytochrome c, mitochondrial apoptosis) (**L**), and APP (amyloid precursor protein, protein homeostasis) (**M**). All correlations were statistically significant (Pearson correlation analysis, *p* < 0.0001). (**N**–**Q**) Correlation analysis between CSF SELENOP and PSAP (**N**), LCAT (**O**), APP (**P**), and CYCS (**Q**). SELENOP showed significant positive correlations with various proteins related to mitochondria, lysosomes, and protein homeostasis (Pearson correlation analysis, *p* < 0.0001).

Based on CSF and plasma proteomic data from the PPMI cohort, we systematically analyzed selected selenoproteins in PD (Figure 1A). CSF GPX3 abundance was significantly lower in the PD group than in controls, while prodromal individuals exhibited intermediate levels (Figure 1B). In contrast, SELENOP and TXNRD1 showed no significant group differences in either CSF or plasma (Figure 1C–E), suggesting that PD may involve a selective alteration in selenium-dependent antioxidant function rather than systemic selenium deficiency, which is a notion consistent with the known brain-priority selenium retention mechanism [31].

Further logistic regression and ROC curve analyses indicated that GPX3 in the CSF has modest yet significant discriminatory capacity for PD, whereas the predictive utility of SELENOP appeared less pronounced (Figure 1F–I). Correlation analysis revealed significant associations between GPX3 and several prototypical pathological markers, including CYCS (mitochondrial apoptosis), PSAP (lysosomal function), LCAT (lipid metabolism regulation), and APP (protein homeostasis) [32] (Figure 1J–M). Notably, although SELENOP did not exhibit significant changes across disease groups, its expression levels were strongly correlated with the aforementioned pathological proteins, suggesting that the selenium transport system might function as an important upstream node in the selenium-dependent antioxidant network, tightly coupled with the core pathological processes of PD (Figure 1N–Q). These correlations support network-level associations among selenium-related proteins and established PD pathological markers.

### 3.2. Preparation Optimization and Physicochemical Characterization of IPP-Se

**Figure 2:**
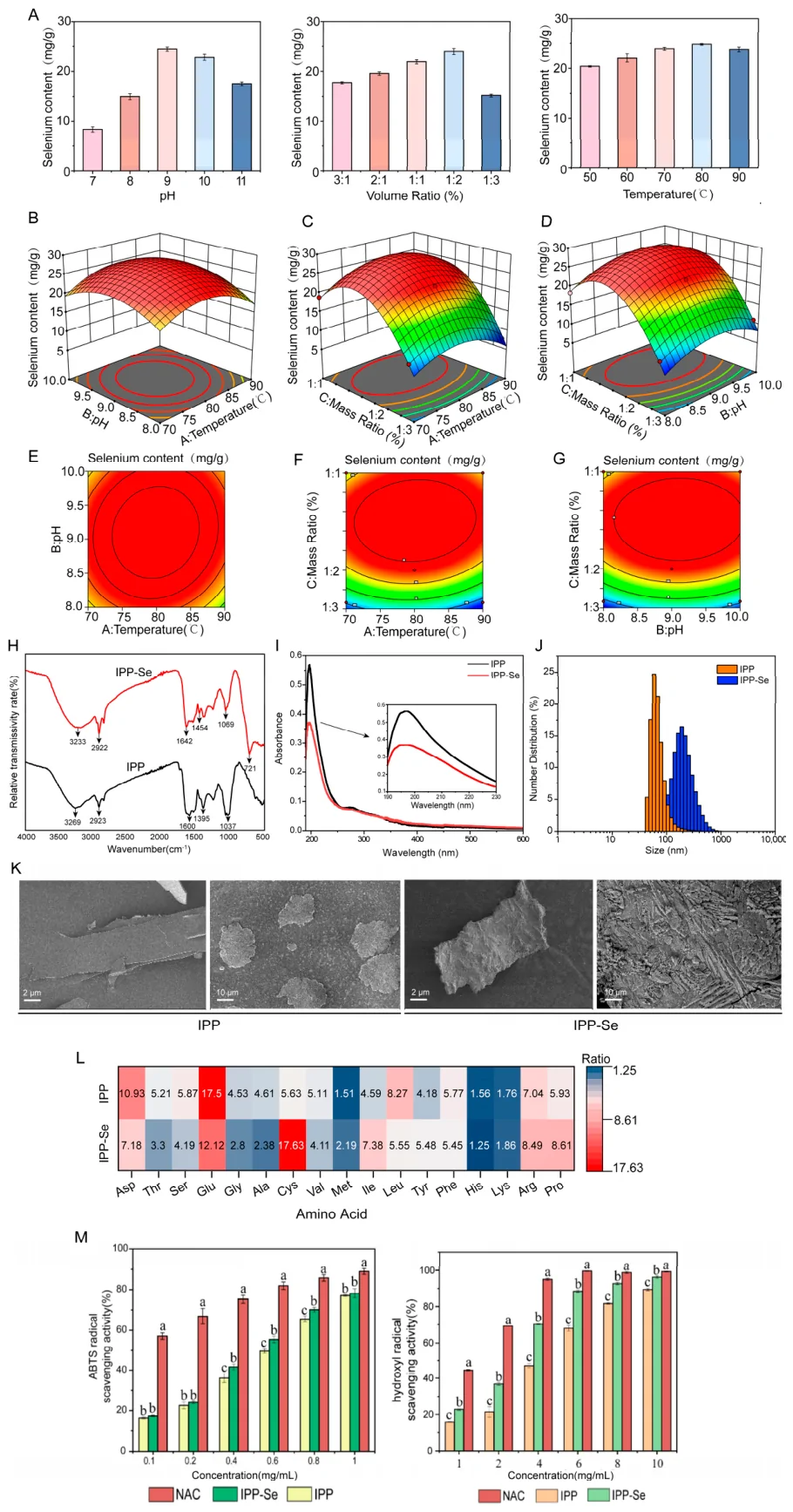
Preparation optimization and physicochemical characterization of IPP-Se. (**A**) Temperature, the volume ratio of the IPP to Na_2_SeO_3_, pH on the selenium chelating activity. (**B**–**G**) Response surface plots (**B**–**D**) and contour plots (**E**–**G**) showing the interactive effects of Temperature, pH, and Volume ratio on the selenium-chelate rate of *Idesia polycarpa* Maxim. cake meal peptide. The color gradients in the response surface and contour plots represent different levels of predicted selenium-chelate rate. Different colors in panel (A) represent different experimental conditions. (**H**) FTIR analysis of IPP and IPP-Se. Compared to IPP, IPP-Se showed distinct changes at multiple characteristic wavenumbers, particularly in bonding regions related to hydroxyl, amino, and selenium groups, suggesting that selenium was successfully introduced and participated in interactions within the IPP molecular structure. (**I**) The formation of complexes of IPP and selenium may lead to the shift/disappearance of pre-existing UV absorbance peaks. The carbonyl or amino group in the amide bond formed a coordination complex with selenium ions. These band changes indicate that IPP-Se was a compound different from IPP. (**J**) DLS particle size distribution analysis of IPP and IPP-Se. IPP-Se exhibited a more concentrated and larger particle size distribution, indicating that selenium modification significantly improved particle dispersion and structural uniformity. (**K**) SEM images of IPP and IPP-Se. IPP presented irregular flake-like or blocky structures, while IPP-Se exhibited a rough, porous, more ordered microscopic morphology, suggesting that the introduction of selenium significantly remodeled the material’s microstructure. (**L**) Amino acid compositions of IPP and IPP-Se. (**M**) ABTS and hydroxyl radical scavenging activities of IPP and crude IPP-Se. Groups denoted by different lowercase letters differ significantly (*p* < 0.05), whereas groups sharing the same letter are not significantly different.

To generate a selenium-associated peptide formulation for subsequent biological evaluation, *Idesia polycarpa* Maxim. cake meal protein-derived peptides (IPP) were reacted with sodium selenite. The optimization began with single-factor tests indicating that pH, volume ratio, and temperature are critical determinants of chelation efficiency (Figure 2A). pH exerted a profound influence, with an optimal value at 9.0. And extreme acidic or alkaline conditions likely weaken inter-peptide interactions, thereby hindering coordination with selenium ions [33]. Regarding precursor concentration, a volume ratio of 1:2 (IPP to Na_2_SeO_3_) yielded the highest incorporation, aligning with reports that excessive ratios result in site saturation [34]. Furthermore, maximum selenium content was achieved at 80 °C, as higher temperatures potentially destabilized chelation bonds [29]. Using RSM, we systematically examined the effects of temperature, pH, and volume ratio on the selenium content in IPP-Se (Figure 2B–G). To refine these interactions, a Box–Behnken design (Tables S1 and S2) identified the optimal preparation region through the regression equation. The selenium content (Y) was expressed as follows: Y = 27.7687 − 0.1917A + 0.8525B + 6.0679C + 0.76AB + 0.9164AC + 0.1511BC − 4.6450A^2^ − 4.0950B^2^ − 10.1166C^2^ (R^2^ = 0.9954). The results showed significant interactions between these parameters, with selenium loading reaching an optimal level of 28.73 mg/g at a temperature of 80.18 °C, a volume ratio of 1:1.32, and a pH of 9.1. This indicates that the formation of IPP-Se depends on multi-factorial synergistic regulation rather than single-factor linear patterns. The pH had a significant effect on the chelation rate, followed by the volume ratio and chelation temperature (Table S3).

After preparation of IPP-Se under the optimized conditions, we first characterized selenium treatment-induced changes in the local chemical environment using spectroscopic analyses. FTIR spectra revealed several differences between IPP and IPP-Se (Figure 2H). The broad band associated with N-H/O-H stretching shifted from 3269 to 3233 cm^−1^, while bands related to the amide and carboxylate environments shifted from 1598 to 1642 cm^−1^ and from 1395 to 1454 cm^−1^, respectively. In addition, the band at 1037 cm^−1^ shifted to 1069 cm^−1^, and a new low-wavenumber feature appeared at approximately 721 cm^−1^. Collectively, these changes indicate that selenium treatment altered the local chemical environments of nitrogen- and oxygen-containing functional groups within the peptide preparation and are consistent with the formation of selenium-associated interactions. However, the FTIR peak shifts alone are insufficient to identify a unique selenium-binding site or establish a specific selenium-peptide covalent-bond configuration. To examine these chemical-environment changes using an independent spectroscopic approach, IPP and IPP-Se were further analyzed by UV-Vis spectroscopy. Consistent with the FTIR findings, the major absorption maximum shifted from 197 to 194 nm following selenium treatment, while the absorption feature near 274 nm was no longer detected (Figure 2I). These spectral changes may reflect alterations in the electronic environments surrounding carbonyl, amino, and aromatic groups, further indicating that IPP-Se possesses spectroscopic properties distinct from those of untreated IPP. Nevertheless, as with FTIR, the UV-Vis changes alone do not define the specific mode of selenium binding. Because alterations in the local chemical environment may also induce conformational rearrangement of the peptide chains, the amide I region of the FTIR spectra was subsequently deconvoluted. The proportion of β-sheet structures increased from 30.49% in IPP to 37.14% in IPP-Se, whereas the α-helical content decreased from 14.12% to 12.31%. These results indicate that selenium treatment was accompanied not only by changes in functional-group environments but also by rearrangement of the peptide secondary structure.

We next examined whether these molecular and conformational changes were reflected at a higher level of assembly using dynamic light scattering. In deionized water, the mean hydrodynamic diameter increased from 79.16 ± 0.44 nm for IPP to 230.60 nm for IPP-Se, while the PDI decreased from 0.55 ± 0.01 to 0.21 ± 0.16 (Figure 2J). Thus, selenium treatment resulted in the formation of larger peptide assemblies with a relatively narrower hydrodynamic size distribution. This observation is consistent with possible interpeptide association or multivalent interactions during IPP-Se preparation, although DLS cannot directly establish a specific intermolecular cross-linking mechanism. Moreover, because the measurements were performed in deionized water, these data do not demonstrate the colloidal stability of IPP-Se in PBS, physiological saline, serum, or other physiologically relevant media. In agreement with the DLS results, SEM imaging further revealed selenium treatment-induced remodeling of the microscopic morphology (Figure 2K). Untreated IPP exhibited relatively smooth and compact flake-like structures, whereas IPP-Se displayed a rougher, more porous, and loosely assembled block-like morphology. These observations indicate that the changes induced by selenium treatment extended beyond local functional-group and secondary-structure alterations to affect the supramolecular organization and microscopic architecture of the peptide material.

Having established that IPP-Se differed from IPP in its spectroscopic, conformational, and morphological properties, we next analyzed the amino acid composition to determine whether the preparation process was accompanied by selective enrichment or retention of particular residues. A total of 20 amino acids were detected (Figure 2L), among which Leu, Ala, Gly, Ile, and Lys were relatively abundant, accounting for 10.55%, 9.87%, 9.37%, 7.85%, and 6.84%, respectively. Following selenium treatment, the relative abundances of several amino acids changed, including increases in Lys, Pro, Met, Ile, and Cys, with Cys reaching 17.63% in IPP-Se. These compositional changes may reflect selective enrichment, differential recovery during precipitation, or structural reorganization during IPP-Se preparation. However, changes in amino acid composition alone cannot independently demonstrate substitution of sulfur by selenium in individual Met or Cys residues. Additionally, to determine whether these physicochemical changes were accompanied by altered functional properties, the in vitro radical-scavenging activities of IPP and IPP-Se were compared. IPP-Se exhibited stronger concentration-dependent antioxidant activity than unmodified IPP (Figure 2M). The IC_50_ values of IPP-Se for ABTS^+^ and hydroxyl-radical scavenging were 0.52 ± 0.02 mg/mL and 2.77 ± 0.01 mg/mL, respectively, whereas those of IPP were 0.60 ± 0.01 mg/mL and 4.23 ± 0.14 mg/mL, respectively. For NAC, the ABTS^+^ scavenging rate at the lowest tested concentration of 0.1 mg/mL was already well above 50%, indicating that its IC_50_ value was below 0.1 mg/mL, while its hydroxyl-radical-scavenging IC_50_ value was 1.23 ± 0.02 mg/mL. These findings indicate that selenium treatment was associated with enhanced in vitro radical-scavenging activity. However, because an IPP plus Na_2_SeO_3_ physical-mixture control was not included, the present results cannot fully distinguish selenium–peptide association-dependent effects from potential additive contributions of the peptide and selenium components.

Taken together, FTIR and UV-Vis analyses first demonstrated changes in the local chemical environment following selenium treatment, while amide I deconvolution further revealed secondary-structure rearrangement. DLS and SEM subsequently showed that these molecular-level changes were accompanied by altered particle assembly and microscopic morphology. Combined with the amino acid composition and in vitro antioxidant results, these data support the conclusion that IPP-Se is a selenium-associated peptide formulation that differs from the original IPP in its chemical environment, conformation, assembly state, and functional properties. Nevertheless, its precise selenium-binding sites, bond configuration, and stability under physiological conditions remain to be established.

### 3.3. Sequential Fractionation and Antioxidant Enrichment of IPP-Se-Derived Peptides

**Figure 3:**
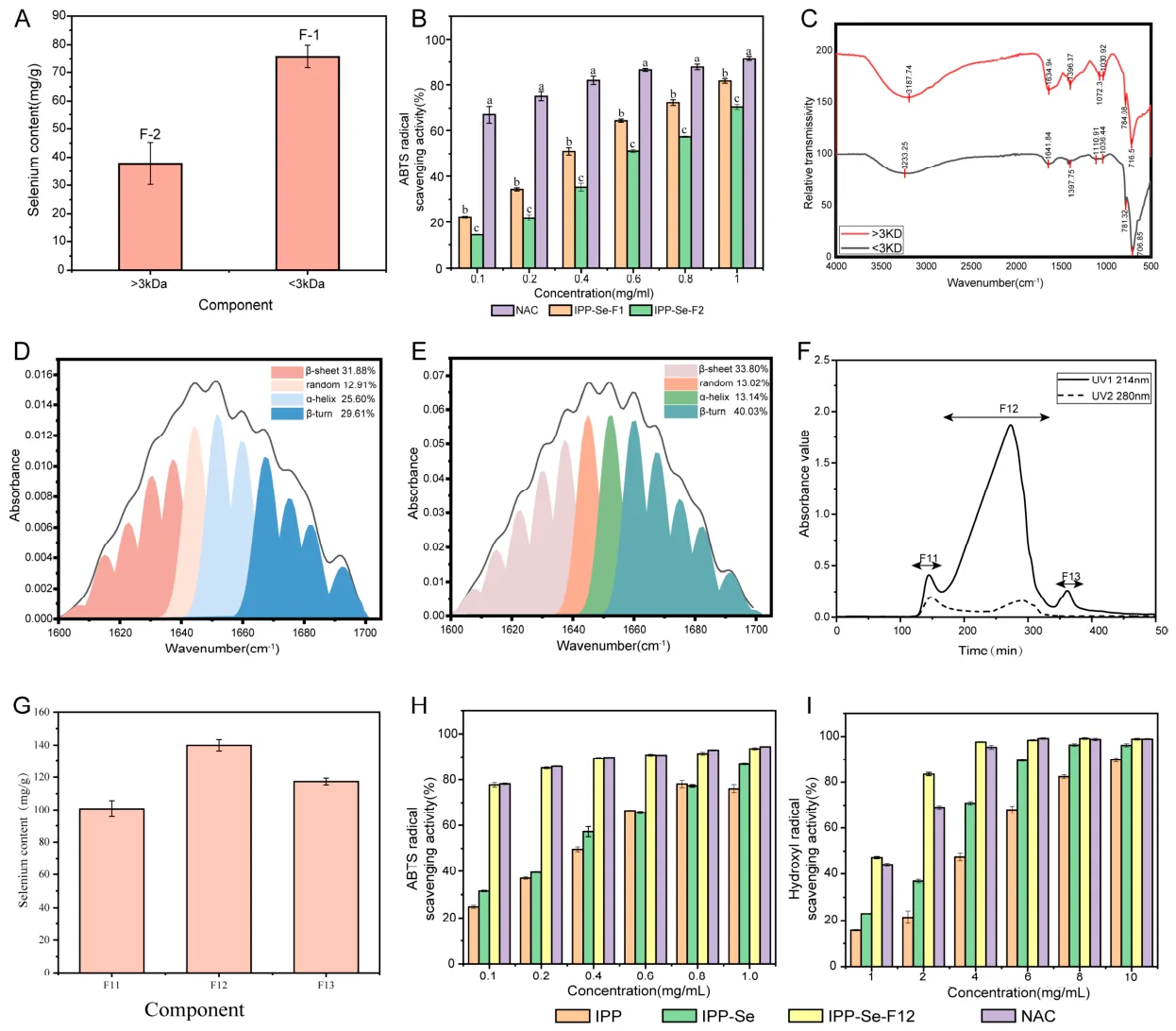
Sequential fractionation, structural characterization, and antioxidant activity of IPP-Se-derived selenium peptides. (**A**) Selenium content of ultrafiltration fractions F1 and F2. (**B**) ABTS radical-scavenging activity of ultrafiltration fractions F1 and F2. Different lowercase letters indicate significant differences between groups (*p* < 0.05). (**C**–**E**) FT-IR spectra and secondary structure composition of ultrafiltration fractions F1 and F2. (**F**) Sephadex gel chromatography elution profile of fraction F1, showing subfractions F11, F12, and F13. (**G**) Selenium content of subfractions F11, F12, and F13 collected from gel filtration. (**H**,**I**) Antioxidant activity of the purified subfraction F12 (purified IPP-Se-F12), which exhibited the highest selenium content and was selected for subsequent mechanistic studies.

Following the physicochemical characterization of the crude IPP-Se preparation, we next sought to identify the peptide fraction in which selenium content and antioxidant activity were preferentially enriched. IPP-Se was first separated using a 3 kDa ultrafiltration membrane, yielding two fractions, designated F1 and F2. Comparison of their selenium contents showed that F1 contained significantly more selenium than F2 (Figure 3A). Consistent with this enrichment, F1 also exhibited stronger concentration-dependent ABTS radical-scavenging activity than F2 (Figure 3B). The parallel enrichment of selenium content and radical-scavenging activity therefore supported the selection of F1 for subsequent purification.

To determine whether the functional differences between the two ultrafiltration fractions were accompanied by structural differences, F1 and F2 were further examined by FTIR spectroscopy and amide I deconvolution. The FTIR spectra revealed several differences between the fractions (Figure 3C). In F1, the N-H/O-H-associated absorption region shifted from approximately 3188 to 3233 cm^−1^, while an amide-associated band shifted from approximately 1635 to 1642 cm^−1^. In addition, a low-wavenumber selenium-associated feature remained detectable after ultrafiltration, with only a small shift from approximately 784 to 781 cm^−1^. The retention of this spectral feature suggests that at least part of the selenium-associated peptide structure persisted during the ultrafiltration procedure. However, this observation does not establish a specific selenium-peptide bond or demonstrate stability under physiological conditions. Amide I deconvolution further showed that F1 and F2 differed in their secondary-structure distributions (Figure 3D,E). The α-helical content of F1 was 25.60%, compared with 13.14% in F2. In contrast, F2 contained relatively higher proportions of random coil and β-turn structures, reaching 13.02% and 40.03%, respectively. These results indicate that ultrafiltration separated peptide populations that differed not only in selenium content and antioxidant activity but also in conformational organization. Together, the compositional, functional, and structural comparisons identified F1 as the fraction most suitable for further purification.

F1 was therefore subjected to Sephadex G-25 gel-filtration chromatography to achieve additional separation according to molecular size. The elution profile monitored at 280 nm resolved F1 into three major subfractions, designated F11, F12, and F13 (Figure 3F). The fractions eluted in the order F11, F12, and F13, consistent with a progressive decrease in apparent molecular size. Measurement of selenium content showed that F12 contained significantly more selenium than F11 and F13 (Figure 3G). On this basis, F12 was selected as the principal purified selenium-associated peptide fraction for subsequent functional and molecular characterization. To confirm that selenium enrichment in F12 was accompanied by retention of antioxidant activity, its ABTS^+^ and hydroxyl-radical-scavenging capacities were evaluated (Figure 3H,I). F12 displayed concentration-dependent activity in both assays and showed stronger radical-scavenging effects than unmodified IPP and the less-purified IPP-Se preparation. In the ABTS^+^ assay, the IC_50_ values of IPP-Se and IPP were 0.32 ± 0.01 mg/mL and 0.40 ± 0.01 mg/mL, respectively. For both F12 and NAC, the ABTS^+^ scavenging rates at the lowest tested concentration of 0.1 mg/mL were already well above 50%; therefore, their IC_50_ values were considered to be below 0.1 mg/mL. Within the tested concentration range, the ABTS^+^ scavenging activity of F12 was comparable to that of the NAC positive control. In the hydroxyl-radical assay, F12 also exhibited strong scavenging activity. The IC_50_ values of IPP, IPP-Se, F12, and NAC were 4.23 ± 0.14, 2.77 ± 0.01, 1.08 ± 0.01, and 1.23 ± 0.02 mg/mL, respectively. F12 showed a lower IC_50_ value than NAC and exhibited stronger hydroxyl-radical-scavenging activity at several concentrations between 1 and 4 mg/mL. The sequential ultrafiltration and gel-filtration procedures progressively enriched a peptide fraction combining relatively high selenium content with strong in vitro radical-scavenging activity. The concordant selenium-content, structural, and antioxidant results identified IPP-Se-F12 as the principal fraction for subsequent LC-MS/MS characterization and molecular docking analysis.

### 3.4. Molecular Composition and Predicted KEAP1-Binding Properties of IPP-Se-F12

**Figure 4:**
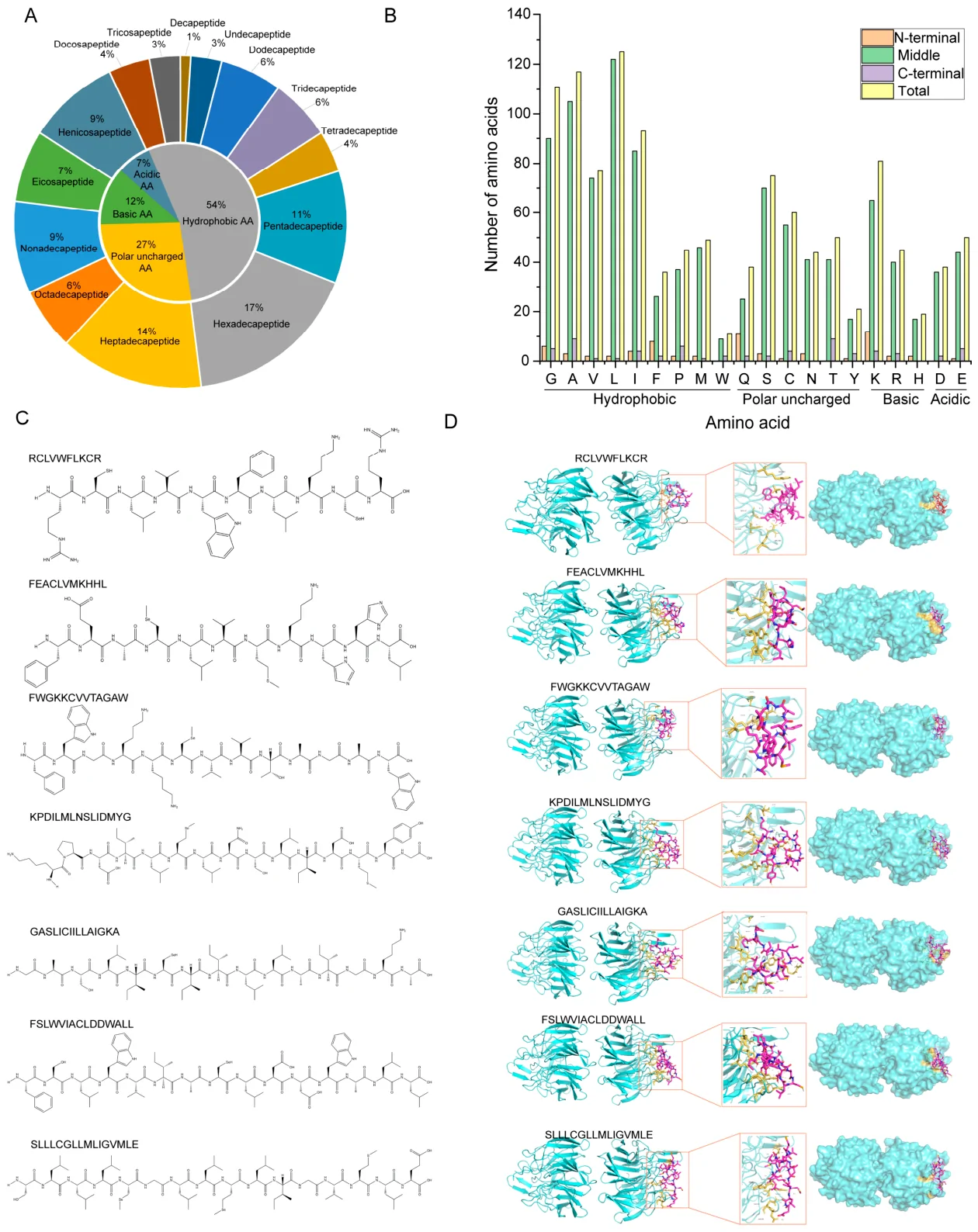
Structural characterization, amino acid composition, and KEAP1-binding properties of IPP-Se-F12. (**A**) Distribution of peptide number (outer ring) and amino acid properties of the constituent amino acids (inner ring) within IPP-Se-F12. (**B**) Composition and distribution of amino acids within IPP-Se-F12. (**C**) Two-dimensional structure of active peptides from IPP-Se-F12 from mass spectrometry analysis. (**D**) Interaction between IPP-Se-F12s and KEAP1 (5WFV) active site molecules. Different colors were used only for visual differentiation among groups.

Following sequential fractionation, which yielded a fraction enriched in both selenium content and radical-scavenging activity, the purified IPP-Se-F12 fraction was further analyzed by LC-MS/MS to characterize its molecular composition. A total of 70 database-assisted selenium-associated peptide assignments were obtained, with peptide lengths ranging from 10 to 23 amino acids and molecular masses ranging from 1321.612 to 2501.182 Da (Figure 4A–C and Table 1). Peptides containing 15–17 amino acids were among the most frequently represented length classes, with putative selenium-containing residue forms including SeCys, SeMet, and MeSeCys. Therefore, IPP-Se-F12 is more appropriately described as a compositionally heterogeneous selenium-associated peptide fraction.

To further examine compositional features potentially related to the antioxidant activity of F12, the amino acids within the identified peptide set were classified according to their physicochemical properties. Hydrophobic amino acids accounted for approximately 54% of all residues, followed by polar uncharged amino acids at approximately 24%, while basic and acidic amino acids accounted for approximately 12% and 7%, respectively (Figure 4A). Hydrophobic residues were also frequently located at both termini of the peptides, accounting for 44.62% of the N-terminal residues and 47.69% of the C-terminal residues (Figure 4B). The enrichment of hydrophobic amino acids, particularly at the N- and C-termini, was consistent with the strong in vitro radical-scavenging activity of IPP-Se-F12 observed in Figure 3.

Given the strong in vitro radical-scavenging activity of IPP-Se-F12, we further investigated whether its candidate selenium-associated peptides might interact with upstream regulators of cellular antioxidant responses. The KEAP1-NRF2 pathway is a classical regulatory axis of cellular antioxidant defense, and the Kelch domain of KEAP1 contains a structurally well-defined peptide-binding pocket that physiologically recognizes the NRF2 ETGE and DLG motifs. Therefore, nine selenium-associated candidate peptides with a PeptideRanker score > 0.5 and a hydrophobic amino acid proportion ≥ 50% were selected from the LC-MS/MS dataset for exploratory peptide–protein molecular docking. The selected peptides ranged from 10 to 16 amino acids in length, while peptide length was not used as a selection criterion. These peptides were not selected based on sequence similarity to the NRF2 ETGE or DLG motifs. Their putative two-dimensional structures and selenium-containing residue annotations are shown in Figure 4C. Docking was performed using the crystal structure of the KEAP1 Kelch domain (PDB ID: 5WFV) as the receptor. The predicted binding energies of the nine candidate peptides ranged from −5.5 to −6.7 kcal/mol (Figure 4D, Tables S4 and S5). Among them, seven peptides were predicted to contact residues located within or adjacent to the KEAP1 ligand-recognition interface, including Arg380, Arg415, and Arg483. These findings therefore support a testable hypothesis that some IPP-Se-F12-derived peptides may interact with the ligand-binding surface of the KEAP1 Kelch domain and potentially influence KEAP1-NRF2 regulation.

### 3.5. Validation of Protective Effects of IPP-Se in the C. elegans PD Model

**Figure 5:**
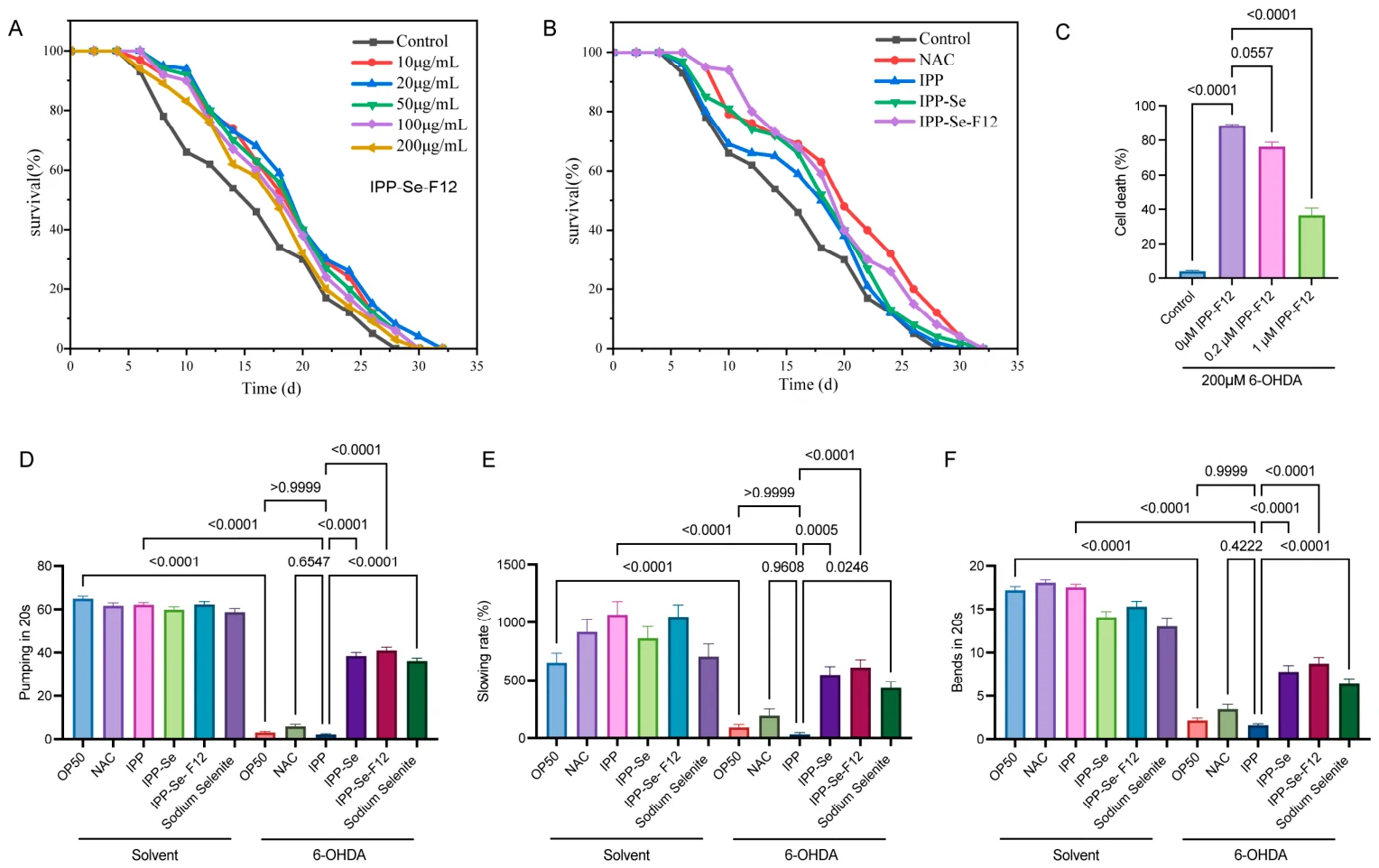
Rescue effects of IPP-Se-F12 on 6-OHDA-induced functional impairment in nematodes, protection of dopaminergic neuron, and prolonged lifespan. (**A**) Effect of IPP-Se-F12 with different concentration on the lifespan of N2 *C. elegans*. (**B**) Effect of IPP, IPP-Se and IPP-Se-F12 on the lifespan of N2 *C. elegans*. (**C**) IPP-Se-F12 supplementation alleviated 6-OHDA-induced cell death in SH-SY5Y cells. Data were analyzed using One-way ANOVA, with significance marked as shown in the figure. (**D**) Analysis of pharyngeal pumping frequency (Pumping in 20 s) in *C. elegans*. The pumping rates were compared between the solvent control group (Solvent) and the 6-OHDA injury group under different treatment conditions. 6-OHDA treatment caused a significant decline in feeding ability, while purified selenopeptide intervention significantly reversed this impairment, showing superior effects compared to crude selenopeptide and even the positive control NAC. (**E**) Food sensing behavior assay (Basal Slowing Response, %). This metric reflects the functional integrity of dopaminergic neurons. 6-OHDA injury resulted in a near-total loss of slowing behavior in the presence of food (indicating impaired dopamine signaling), while purified selenopeptide treatment significantly restored the slowing ratio, suggesting a significant protective effect on dopaminergic neurons. (**F**) Locomotion analysis (Bends in 20 s). The number of body bends within 20 s was counted to assess motor function. Purified selenopeptide significantly improved 6-OHDA-induced motor retardation, restoring locomotion levels close to the normal control group. Data were analyzed using One-way ANOVA, with significance marked as shown in the figure.

To evaluate the biological protective effects of IPP-Se-F12, we first examined its influence on the lifespan of wild-type N2 *C. elegans* to determine an appropriate treatment concentration for subsequent nematode experiments [35]. Within the range of 10–20 μg/mL, IPP-Se-F12 prolonged the mean lifespan of the worms in a concentration-dependent manner. However, the lifespan-extending effect gradually declined at concentrations above 20 μg/mL, resulting in an overall bell-shaped concentration–response relationship (Figure 5A). The greatest effect was observed at 20 μg/mL, which extended the mean lifespan by approximately 21.29% compared with the untreated control. Therefore, 20 μg/mL was selected as the working concentration for subsequent nematode experiments. On this basis, we compared the lifespan-extending effects of different peptide preparations at the same treatment concentration. IPP-Se-F12 produced a greater increase in the mean lifespan of N2 worms than either unmodified IPP or crude IPP-Se (Figure 5B), suggesting that sequential fractionation and purification may have enriched selenium-associated peptide components with stronger biological activity. In addition, none of the peptide preparations shortened worm lifespan under the present experimental conditions, indicating that no obvious lifespan-related toxicity was observed at the selected concentration.

After determining an appropriate treatment concentration for the nematode experiments and initially evaluating the overall biological effects of IPP-Se-F12, we further examined its protective activity using a 6-OHDA-induced SH-SY5Y cell injury model. Exposure to 6-OHDA significantly increased SH-SY5Y cell death, whereas IPP-Se-F12 treatment markedly attenuated this effect (Figure 5C). These results indicate that IPP-Se-F12 can reduce 6-OHDA-induced neuronal cell injury, providing cellular-level evidence for its potential neuroprotective activity.

We subsequently used a 6-OHDA-induced *C. elegans* model of dopaminergic neurotoxicity to further evaluate the functional protective effects of IPP-Se-F12 at the whole-organism level. Exposure to 6-OHDA significantly impaired multiple physiological and behavioral functions in the worms. In the pharyngeal pumping assay, 6-OHDA-treated worms exhibited a marked reduction in pumping frequency, indicating impaired feeding function (Figure 5D). IPP-Se-F12 treatment significantly attenuated this reduction and produced a stronger recovery than crude IPP-Se.

Because pharyngeal pumping primarily reflects the general physiological condition and feeding ability of the worms, we further assessed the basal slowing response to evaluate dopamine-dependent behavioral function. Under normal conditions, worms reduce their locomotor speed when encountering food, and this behavioral response depends on intact dopaminergic signaling. Following 6-OHDA exposure, the basal slowing response was almost completely abolished, indicating severe impairment of dopamine-dependent behavioral function (Figure 5E). In contrast, IPP-Se-F12 treatment significantly restored the basal slowing response, supporting its ability to alleviate 6-OHDA-induced dopaminergic functional impairment. However, because the basal slowing response is a behavioral measure, this result reflects the preservation of dopamine-dependent function and does not independently demonstrate direct protection of dopaminergic neuron structure or number. A similar protective trend was observed in the locomotion assay. Exposure to 6-OHDA significantly reduced the number of body bends within 20 s, indicating marked impairment of motor function (Figure 5F). IPP-Se-F12 treatment significantly increased body-bend frequency and restored locomotor performance toward the level of the solvent control group. Compared with crude IPP-Se, IPP-Se-F12 produced a greater improvement, further supporting the higher biological protective activity of the purified F12 fraction. These findings support the functional protective effects of IPP-Se-F12 at both the cellular and whole-organism levels.

### 3.6. Transcriptomic Responses Associated with IPP-Se-F12 Intervention

**Figure 6:**
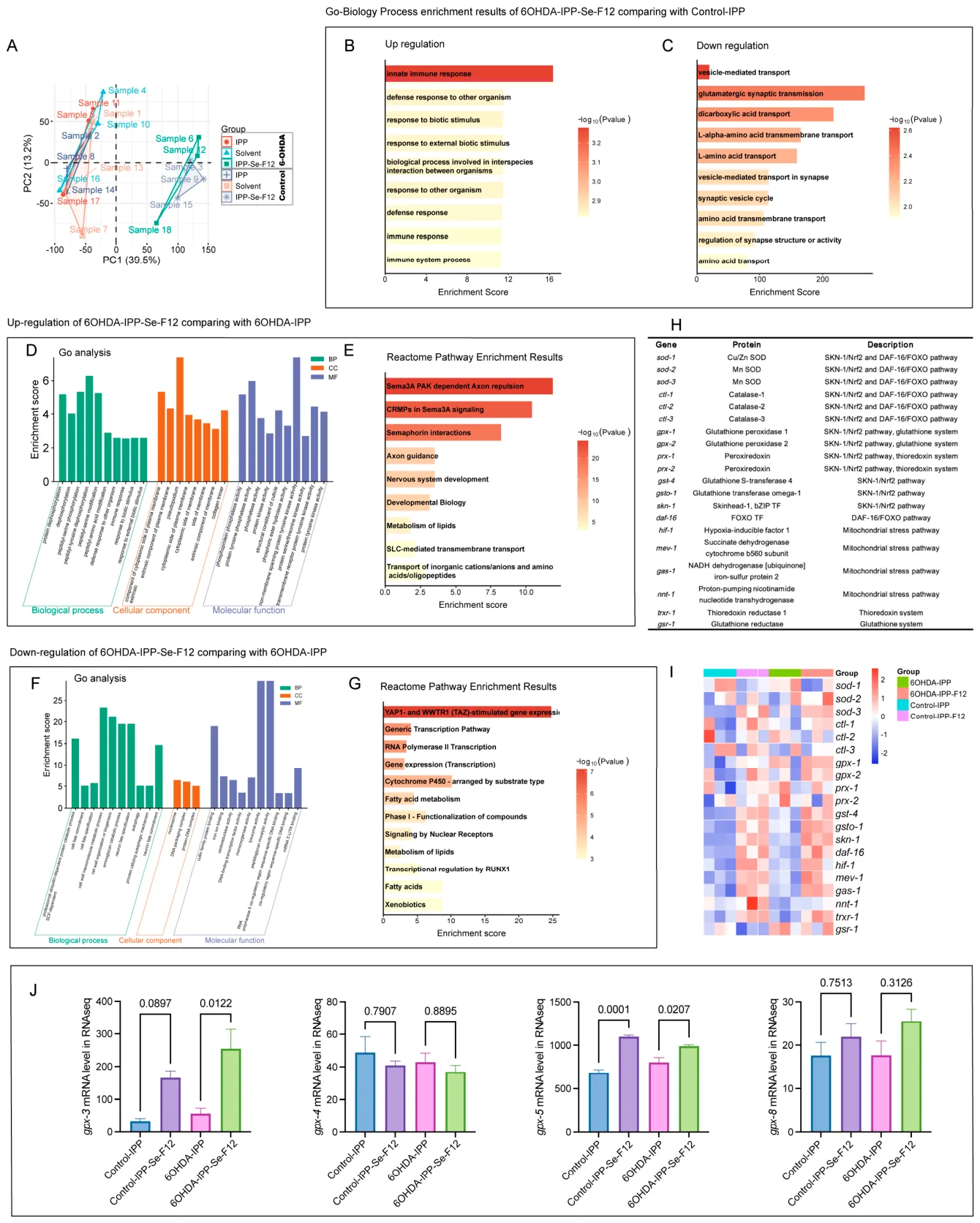
Purified selenopeptides alleviate neurotoxicity via regulation of antioxidant and synaptic pathways. (**A**) PCA plot of transcriptomic data. This displays the overall distribution of gene expression profiles across different treatment groups (Solvent, 6OHDA-IPP), 6OHDA-IPP-Se-F12, etc.). The spatial distance between samples reflects the similarity of their transcriptomic composition. (**B**,**C**) GO enrichment analysis of differentially expressed proteins between the 6-OHDA injury group (6OHDA-IPP) and the Control group (Control-IPP). (**B**) shows that upregulated proteins are mainly enriched in inflammation-related processes such as innate immune response and defense response; (**C**) shows that downregulated proteins are mainly concentrated in neural function-related pathways such as vesicle-mediated transport in synapse and glutamatergic synaptic transmission. (**D**,**E**) Functional enrichment analysis of upregulated proteins in the purified selenium peptide treatment group (6OHDA-IPP-Se-F12) compared to the injury group (6OHDA-IPP). (**D**) shows GO analysis covering Biological Process (BP), Cellular Component (CC), and Molecular Function (MF); (**E**) shows Reactome pathway enrichment analysis. (**F**,**G**) Functional enrichment analysis of downregulated proteins in the purified selenium peptide treatment group (6OHDA-IPP-Se-F12) compared to the injury group (6OHDA-IPP). (**F**) shows GO analysis; (**G**) shows Reactome pathway analysis. (**H**) List of key differentially expressed proteins and their functional annotations. Proteins playing core roles in antioxidant defense and mitochondrial homeostasis are listed, including SKN-1/Nrf2 pathway and DAF-16/FOXO pathway-related proteins (e.g., SODs, Catalases, GPXs) and members of the glutathione system. (**I**) Heatmap of key antioxidant and stress defense proteins. Colors represent the relative protein expression levels (Z-score), with red indicating high expression and blue indicating low expression. (**J**) The bar chart shows the expression levels of *gpx-3*, *gpx-4*, gpx-5, and *gpx-8* in RNA-seq, which are homologous to the human *GPX3* gene. Additionally, peptide treatments were administered at 20 μg/mL, and the worms were exposed to 10 mM 6-OHDA for 1 h. OP50 denotes the untreated control group (only with solvent) maintained on the standard *Escherichia coli* OP50 food strain.

To explore the molecular processes potentially involved in the ability of IPP-Se-F12 to alleviate 6-OHDA-induced functional impairment, RNA-seq analysis was performed on *C. elegans* from the different treatment groups (Figure 6). Principal component analysis revealed clear differences in the overall gene-expression profiles among the groups (Figure 6A). In particular, the IPP-Se-F12-treated group was separated from the 6-OHDA injury group along the PC2 axis, indicating that IPP-Se-F12 intervention was associated with substantial transcriptomic remodeling. Meanwhile, compared with the solvent control group, 6-OHDA exposure upregulated genes related to immune and defense responses while downregulating genes associated with synaptic transmission and vesicle-mediated transport (Figure 6B,C). These transcriptional changes were consistent with the impairments in feeding, locomotion, and dopamine-dependent behavior observed in Figure 5, thereby providing molecular support for the successful establishment of the 6-OHDA injury model.

We subsequently focused on the comparison between the IPP-Se-F12-treated 6-OHDA group and the untreated 6-OHDA group to characterize the transcriptional responses associated with IPP-Se-F12 intervention. GO biological-process enrichment analysis identified peptidyl-tyrosine dephosphorylation and dephosphorylation as significantly enriched terms. Consistently, GO molecular-function analysis further identified enrichment of protein tyrosine phosphatase activity and phosphoprotein phosphatase activity (Figure 6D,E). Together, these findings suggest that regulation of protein phosphorylation status may represent an important component of the transcriptional response associated with IPP-Se-F12 intervention. In addition, autophagy-related biological processes were significantly enriched among the genes downregulated following IPP-Se-F12 treatment (Figure 6F,G), suggesting that IPP-Se-F12 intervention may be associated with the transcriptional regulation of autophagy-related genes. Reactome pathway analysis further showed that IPP-Se-F12-responsive genes were enriched in pathways related to axon guidance and neuronal function regulation, including the annotated Sema3A PAK-dependent axon-repulsion pathway (Figure 6H). These findings suggest that IPP-Se-F12 intervention may influence transcriptional programs associated with neuronal guidance, cytoskeletal regulation, and the maintenance of neuronal function.

Given the strong in vitro radical-scavenging activity of IPP-Se-F12, we assessed the expression profiles of selected antioxidant effectors (Figure 6I). Heatmap analysis showed increased expression of several antioxidant genes, including *sod-1*, *gpx-1*, and *gst-4*, following IPP-Se-F12 treatment. IPP-Se-F12 treatment was also associated with increased expression of *gpx-3*, the *C. elegans* ortholog of human GPX3 [36]. Among other GPX-family genes, *gpx-4*, *gpx-5*, and *gpx-8* showed treatment-associated expression patterns (Figure 6J). These findings indicate that IPP-Se-F12 treatment is associated with transcriptional changes in GPX-related antioxidant responses, although direct mechanistic validation is still required.

Overall, compared with the untreated 6-OHDA injury group, IPP-Se-F12 treatment was associated with two major categories of transcriptional responses: those involving protein dephosphorylation, axon guidance, and neuronal function-related processes, and those involving oxidative-stress responses and altered expression of glutathione peroxidase family genes.

## 4. Discussion

The relationship between Se and PD has long been framed primarily as a systemic attenuation of antioxidant capacity, precipitated by diminished peripheral Se levels or chronic dietary insufficiency [37,38]. This view has been supported by multiple animal models and epidemiological studies, which show that selenium supplementation attenuates dopaminergic neuron damage and ameliorates motor dysfunction [39]. However, increasing evidence indicates that the central nervous system exhibits a highly prioritized homeostatic resilience [9]. Even under conditions of peripheral Se deficiency, the brain can maintain its basal selenium supply through tightly regulated transport and redistribution mechanisms [40]. Consequently, the conventional ‘selenium deficiency’ model of PD pathogenesis appears reductive. By integrating large-scale CSF and plasma proteomic datasets from the PPMI cohort, our findings suggests that selenium-associated alterations in PD may be compartment-specific rather than uniformly systemic. In particular, the reduction in CSF GPX3 identifies impaired GPX3-related extracellular antioxidant defense as a potential molecular feature of PD [13,14]. This result aligns with recent clinical evidence demonstrating that peripheral Se concentrations in PD patients often remain indistinguishable from healthy controls, while CSF Se levels are significantly depleted [11]. It provides a clinically relevant context for evaluating interventions that may influence glutathione-peroxidase-related antioxidant responses. The correlation between SELENOP and lysosomal (PSAP) and mitochondrial (CYCS) markers observed in our PPMI analysis is biologically plausible. Recent evidence demonstrates that SELENOP deficiency in microglia downregulates extracellular vesicle biogenesis machinery and is accompanied by increased lysosomal activity and altered lipid metabolism [41]. Furthermore, selenium transport via the SELENOP-LRP8/ApoER2 axis is essential for maintaining mitochondrial antioxidant defenses, and dysfunction of this transport system has been linked to mitochondrial dysfunction and neurodegeneration [42]. These findings provide a mechanistic context for the association between SE-LENOP and both lysosomal and mitochondrial pathways observed in our correlation analysis.

Selenium nutrition is characterized by a relatively narrow physiological window, as deficiency can cause multisystem consequences, whereas excessive exposure may increase the risk of toxicity [43]. Plant-derived selenium-enriched peptides represent a compelling alternative to conventional interventions, integrating the superior bioavailability of organic selenium with the multifaceted bioactivity of functional peptides [44]. However, their clinical translation is currently obstructed by protracted production cycles and suboptimal yield. To develop a selenium-based formulation derived from plant peptides, peptides sourced from defatted *Idesia polycarpa* Maxim. cake meal were reacted with sodium selenite under optimized conditions. Subsequent stepwise separation via ultrafiltration and gel filtration chromatography yielded IPP-Se-F12, a selenium-enriched peptide fraction exhibiting distinct physicochemical properties compared to the unmodified IPP. FTIR and UV-Vis analyses indicated alterations in the local chemical environments of specific functional groups within the peptides following selenium treatment. These spectral changes coincided with a redistribution of secondary structure proportions, an increase in hydrodynamic particle size, a reduction in PDI, and significant modifications in microscopic morphology. Collectively, these findings suggest that selenium treatment influences both peptide chain conformation and higher-order assembly states.

Stepwise fractionation further enriched both selenium content and in vitro antioxidant activity. The low-molecular-weight F1 fraction exhibited higher selenium content and superior ABTS radical scavenging activity compared to F2, and was therefore selected for subsequent purification. Subsequent separation of F1 by Sephadex G-25 gel filtration chromatography yielded the F12 fraction, which displayed the highest selenium content along with potent ABTS and hydroxyl radical scavenging activities. LC-MS/MS analysis further revealed that IPP-Se-F12 is a compositionally heterogeneous peptide fraction containing multiple putative selenium-associated peptides. These peptides varied in amino acid sequence, molecular mass, and the forms of presumed SeCys, SeMet, and MeSeCys residues. Notably, the identified peptides exhibited a high proportion of hydrophobic amino acids, predominantly distributed at the N- and C-termini. Such compositional characteristics may facilitate interactions between the peptides and free radicals or hydrophobic molecular environments, consistent with the robust in vitro radical scavenging activity observed for the F12 fraction [45].

Integrating the cellular, whole-organism, and transcriptomic findings, the protective effects of IPP-Se-F12 may involve both direct radical scavenging and modulation of endogenous stress responses and neurological-function-related pathways. Selenium-dependent redox regulation may also intersect with programmed cell death and secondary tissue injury. For example, in selenium-deficient chicken skeletal muscle, reduced SELENOT expression impairs mitochondrial respiratory-chain function, increases mitochondrial reactive oxygen species, disrupts glucose and NADPH metabolism, and promotes cysteine/disulfide stress, ultimately leading to disulfidptosis and muscle atrophy [46]. Similarly, selenoprotein S deficiency in a mouse model of ulcerative colitis enhanced epithelial oxidative stress, necroptosis, inflammatory signaling, and tight-junction disruption, whereas selenium supplementation alleviated colon injury [47]. Although these findings cannot be directly extrapolated to dopaminergic neurons or nematode behavior, they provide comparative evidence that selenium-dependent regulation can interrupt interconnected oxidative, metabolic, inflammatory, and regulated-cell-death pathways. Notably, IPP-Se-F12 treatment upregulated *gpx-3* and other GPX-family genes, conceptually paralleling the reduced GPX3 abundance observed in the cerebrospinal fluid of patients with PD. However, the human data reflect protein abundance in cerebrospinal fluid, whereas the nematode data represent gene-expression changes, and this cross-species relationship requires further validation. Overall, these findings suggest that both antioxidant activity and regulation of endogenous defense systems may contribute to the protective effects of IPP-Se-F12, although the implicated pathways remain candidate mechanisms that require further investigation.

However, several limitations should be acknowledged. IPP-Se-F12 is a compositionally heterogeneous peptide fraction, and the precise selenium-binding sites and bond configurations of the putative SeCys-, SeMet-, and MeSeCys-containing peptides remain unresolved. In addition, because a comprehensive species-specific proteome database for *Idesia polycarpa* is currently unavailable, the LC–MS/MS data were searched against UniProtKB entries assigned to the broader taxonomic group Flacourtieae. Consequently, some peptide-spectrum matches may correspond to homologous proteins from closely related taxa, and the exact parent proteins of the identified peptides in I. polycarpa cannot be assigned unambiguously. Critical pharmacokinetic and blood–brain barrier penetration data are currently lacking for IPP-Se-F12. Its stability has been assessed only under limited in vitro conditions, while gastrointestinal and serum stability, cellular uptake, systemic selenium exposure, pharmacokinetics, tissue distribution, and blood–brain barrier penetration have not been determined. In addition, the absence of a physical mixture of unchelated IPP and sodium selenite as a control prevents us from conclusively distinguishing between effects dependent on selenium–peptide association and simple additive effects of the individual components. We have therefore refrained from attributing the enhanced activity specifically to chelation. The current protective evidence is also limited to SH-SY5Y cells and *C. elegans* and has not yet been validated in mammalian PD models. Moreover, the pathways suggested by RNA-seq and molecular docking remain associative or computationally predicted and lack direct validation at the protein, enzymatic, and functional levels. The FTIR-derived secondary-structure proportions represent ensemble-averaged characteristics of the heterogeneous IPP and IPP-Se preparations and were not imposed as structural constraints on the individual peptides during docking. Because the docking calculations used Chem3D-generated starting conformations and did not exhaustively sample the conformational ensembles of the flexible peptides, the docking results should be interpreted as exploratory predictions. Future studies should therefore define the chemical structures and selenium-binding sites of the active peptides, assess their stability under physiological and gastrointestinal conditions, and systematically evaluate dose–response relationships, repeated-dose safety, absorption, distribution, metabolism, and brain exposure in mammalian PD models. Targeted experiments using genetic perturbation, direct binding assays, protein and enzyme measurements, and neuronal imaging will also be required to validate GPX3-related antioxidant regulation, KEAP1-NRF2 signaling, phosphatase activity, autophagic flux, and dopaminergic neuronal integrity. These studies will determine whether the observed protective effects can be developed into a chemically defined, mechanistically supported, and biologically safe selenium–peptide intervention.

## 5. Conclusions

This study identified a selective reduction in CSF GPX3 abundance in PD and developed a selenium-enriched peptide fraction, IPP-Se-F12, from *Idesia polycarpa* Maxim. cake meal protein-derived peptides. Following sequential fractionation and purification, IPP-Se-F12 exhibited relatively high selenium content and strong in vitro radical-scavenging activity. It also alleviated 6-OHDA-induced cellular injury and functional impairment in *C. elegans* and was associated with transcriptional responses involving antioxidant defense, protein dephosphorylation, axon guidance, and neuronal function-related processes. These findings suggest that selenium-enriched peptides may provide a potential strategy for reinforcing redox defense in PD-related experimental models. Nevertheless, IPP-Se-F12 remains a chemically heterogeneous peptide fraction, and its precise selenium-binding structures, pharmacokinetics, safety, brain exposure, and mechanisms of action require further validation in mammalian models before any translational conclusions can be drawn.

## Figures and Tables

**Table 1:** Secondary structure contents of Amide I band for peptides from *Idesia polycarpa* Maxim. cake meal and corresponding Se-chelated peptides.

Sample	β-Sheet	Random Coil	α-Helix	β-Turn
IPP	30.49%	12.94%	14.12%	42.45%
IPP-Se	37.14%	12.43%	12.31%	38.12%

## Data Availability

The data generated in this study are openly available in Zenodo at https://doi.org/10.5281/zenodo.21881450, reference number 21881450. The PPMI data analyzed in this study are available from the Parkinson’s Progression Markers Initiative database (https://www.ppmi-info.org/access-data-specimens/download-data) on 29 July 2024, subject to the PPMI data-access requirements.

## Supplementary Materials

The following supporting information can be downloaded at https://www.mdpi.com/article/10.3390/cells15181628/s1: Table S1: Box–Behnken experimental factors and levels; Table S2: Optimal preparation conditions for IPP-Se; Table S3: Analysis of variance for the second-order polynomial model; Table S4: Identification of selenium-enriched peptide sequences from purified IPP-Se; Table S5: Binding affinity and interaction sites between KEAP1 (PDB ID: 5WFV) and IPP-Se predicted by molecular docking.
