# Supplementary material for: Bioactive Selenium Peptides Rescue Dopaminergic Neurodegeneration via Coordinated Signal Remodeling and Redox Reinforcement
Source: Cells. 2026 Sep 8;15(18):1628. doi: 10.3390/cells15181628 (PMC13605311; doi:10.3390/cells15181628)
Supplement: Supplementary file 1 [file cells-15-01628-s001.zip › cells-4525670-supplementary.pdf]

cells

MDPI

Article

Bioactive Selenium Peptides Rescue Dopaminergic Neurodegeneration via Coordinated Signal Remodeling and Redox Reinforcement

Xue Hou [1,2], Lin Luo [1,2], Ruojia Li [1,2], Yuying Li [1,2], Zhiyong Wang [1,2,*] and Yangyang Wu [3,*]

1 Hubei Engineering Research Center of Selenium Food Nutrition and Health Intelligent Technology, Hubei Minzu University, Enshi 445000, China
2 College of Biological and Food Engineering, Hubei Minzu University, Enshi 445000, China
3 School of Life Sciences, Westlake University, Hangzhou, 310000, China
* Correspondence: wangzhiyong@hbmzu.edu.cn (Z. Wang), wyy47465842@163.com (Y. Wu)

Table S1. Box-Behnken experimental factors and levels.

Factor	Level		
	**-1**	**0**	**1**
A: Temperature	70	80	90
B: Mass Ratio	1：3	1：2	1：1
C: pH	8	9	10

Table S2. The optimal preparation method for IPP-Se.

Temperature (°C)	80.18
pH	9.1
Mass Ratio	1:1.32
Selenium Content Achievable（mg/g）	28.73

Table S3. Analysis of variance for the second-order polynomial model.

Variance Model	Sum of squares	df	Mean Square	F-value	p-value
Model	475.11	9	52.91	167.72	< 0.0001
A-Temperature	0.2744	1	0.2744	0.8718	0.3815
B-pH	5.43	1	5.43	117.25	0.0043
C-Mass Ratio	228.12	1	189.15	724.76	< 0.0001
AB	2.31	1	2.31	7.34	0.0302
AC	3.43	1	3.43	10.89	0.0131

BC	0.0932	1	0.0932	0.2960	0.6033
A2	90.85	1	90.85	288.63	< 0.0001
B2	70.61	1	70.61	224.32	< 0.0001
C2	176.31	1	176.31	560.15	< 0.0001
Residual	2.20	7	0.3148		
Lack of Fit	1.11	3	0.7344		
Pure Error	0.0000	4	0.0000		
Cor Total	477.32	16			
	R^2=0.9954		R^2adj=0.9894		

Table S4. Identification of Se-enriched peptide sequences from purified Se-IPP.

Number	Sequence	Length	Speciation of organic Se	Mass	Hydrophobic amino acid ratio (%)
1	RCLVWFLKCSeR	10	C9(S-Se)	1371.677416	50
2	FEACSeLVMKHHL	11	C4(MeSeCyS)	1389.59868	54.55
3	KKMMSeDDFKNQK	11	M4(S-Se)	1476.641161	27.27
4	KLPMSeLYQEKIIE	12	M4(S-Se)	1552.764818	50
5	YAIAGKKGECSeIY	12	C10(MeSeCyS)	1377.637821	33.33
6	HRSLYSEICSeKAI	12	C9(MeSeCyS)	1481.723026	33.33
7	KSRGVSFEIYRCSe	12	C12(MeSeCyS)	1506.710422	25
8	FWGKKCSeVVTAGAW	13	C6(MeSeCyS)	1514.725773	53.85
9	LGITNCSeGVEAIVA	13	C6(MeSeCyS)	1321.611864	53.85
10	IGIICSeVAEGWKQT	13	C5(S-Se)	1465.719164	46.15
11	QSICSeILTLNPNTT	13	C4(MeSeCyS)	1479.712253	38.46
12	LPCSeTDVDLINIQLK	14	C3(S-Se)	1632.815342	50
13	QSVKRLMTFCSeQRRY	14	C10(S-Se)	1863.926853	28.57
14	QTPRKCSeLNIMEQQK	14	C6(S-Se)	1764.834642	28.57
15	FSLWVIACSeLDDWALL	15	C8(S-Se)	1812.834642	73.33
16	GASLICSeIILLAIGKA	15	C6(S-Se)	1503.827074	66.67
17	KPDILMSeLNSLIDMYG	15	M6(S-Se)	1786.8129	53.33

18	FNCSePSVIHYTSIAAT	15	C3(MeSeCyS)	1685.767795	46.67
19	LDLIFSLCSeRSKVEDI	15	C8(MeSeCyS)	1812.87833	46.67
20	EEPAKVEAAYTMSeNKR	15	M12(S-Se)	1784.795444	40
21	QAFFKHGDKEALRSCSe	15	C15(S-Se)	1784.795444	33.33
22	KSTCSeHITSYIELRLC	15	C4(S-Se)	1814.83939	33.33
23	NPWIALRACALCSeLPAI	16	C12(S-Se)	1772.877489	75
24	SLLLCSeGLLMSeLIGVMLE	16	C5(MeSeCyS); M9(S-Se)	1827.855442	68.75
25	FALMSeSLLGASIMPHNF	16	M4(S-Se)	1796.849277	68.75
26	QKLAEMSeEVAGAKKALQ	16	M6(S-Se)	1762.89072	50
27	KGKNTCSePIDVILLRNP	16	C6(MeSeCyS)	1842.934727	43.75
28	NAGKSTLLNAMSeAKcVG	16	C14(MeSeCyS)	1655.75571	43.75
29	KGEILMLNIGSMSeSSGA	16	M12(S-Se)	1655.75571	43.75
30	ANKTIVIEHCSeKQCNAF	16	C10(MeSeCyS)	1880.890842	37.5
31	SRLLHCSeVVVGGGPTGV	16	C6(S-Se)	1598.775303	37.5
32	GMALCKLCSeKQRQKSNG	16	C8(S-Se)	1812.839145	25
33	QKREKFMSeNKEKSDALW	16	M7(S-Se)	2086.018406	25
34	KKEEKKKSQKCSeSLLFG	16	C11(S-Se)	1929.025425	18.75
35	RMLVYLKYCSeFLGLAFPP	17	C9(S-Se)	2079.044834	64.71
36	IPVDIIHMSeLDACIEIPQ	17	M8(S-Se)	1967.932957	64.71
37	PTPGAVFTSSMICSeEATI	17	C13(MeSeCyS)	1786.8129	52.94
38	NLASAARNIIMSeEKNSeQLE	17	M14(S-Se)	1995.96439	52.94
39	RSYIMSeRIIASFSGQCVP	17	M5(S-Se)	1975.930956	47.06
40	HAHVLCVGLEQKAMSeVQS	17	M14(S-Se)	1897.909349	47.06
41	LRIFSRDCSeFAETAINTI	17	C8(SeCys2)	2152.907125	47.06
42	KAKVCSeIDVIVQRLGDAT	17	C5(MeSeCyS)	1890.98563	47.06
43	KNQIDCELLGSCSeSALLT	17	C12(MeSeCyS)	1869.859653	35.29
44	FDMSeGFRDNASVVGNGER	17	M3(S-Se)	1918.768697	35.29
45	KPLNAQPPMLFRCSeNQIAP	18	C13(S-Se)	2086.018406	61.11
46	QGKVNKPVVAWVSGTCSeAT	18	C16(MeSeCyS)	1906.953146	44.44

https://doi.org/10.3390/xxxxx

47	SKTTLLCSeVAWGGGFHVHD	18	C7(S-Se)	1975.931186	38.89
48	FDGKKLTESIKSRMSeQNEA	18	M14(S-Se)	2129.995029	27.78
49	KVPIAVALSCSeLVGMSeTAGVA	19	C10(S-Se)	1863.926853	68.42
50	AMLGRVGALEMGGACSeVVAK	19	C15(S-Se)	1880.890842	57.89
51	VFLGGDSSGANIAHNLAMSeA	19	M18(S-Se)	1892.823093	52.63
52	CSeGNLVPAEVQIDFSSVRHL	19	C1(MeSeCyS)	2145.998813	47.37
53	GNVEDGMTKRGAAMSeVQPLP	19	M14(S-Se)	2018.902012	47.37
54	QTAKAIALECSeGIISSGADT	19	C10(S-Se)	1896.916965	42.11
55	PNTVCSePYWLRQLLFYATSLL	20	C5(MeSeCyS)	2460.238376	55
56	FVLTATLGGMSeFLCSeGANDLIT	20	M10(S-Se); C13(S-Se)	2152.907125	50
57	INDIGVISCSeHKVGKIYPASL	20	C9(MeSeCyS)	2189.104391	45
58	MPPGSAIPGGYNPMMSeGSGGY	20	M15(S-Se)	2004.762607	40
59	QMSeSGRAGRRGKDERGICIIM	20	M2(S-Se)	2282.128025	30
60	QPAEKVASLGSDVPMSeDRAAQP	21	M15(S-Se)	2215.048801	52.38
61	GTATFLTMSeAYILAVNASILAD	21	M8(S-Se)	2204.080465	51.74
62	LGVSSFTHLIAGCSeVPLFFSSA	21	C13(MeSeCyS)	2215.048801	47.62
63	QKTLLNLIGMSeLGNESAPRHLS	21	M10(S-Se)	2340.212789	42.86
64	AVQGAVSGRCILNRDTGGVICSe	21	C21(S-Se)	2137.003757	38.1
65	LGTSLIDMSeYLKCGRIDEGLNA	21	M8(S-Se)	2330.099169	38.1
66	MELIICSePRDCKIKMVASAVTCE	22	C6(S-Se)	2501.182041	50
67	GQAPARQAALGAGIPNSVVCSeTT	22	C20(S-Se)	2129.995029	50
68	KAEGVELMIIIMSeKQKKLAYGSA	22	M12(S-Se)	2469.25687	50
69	GEGEVLDLVVIGCSeGPAGLALAAE	23	C13(MeSeCyS)	2215.048801	56.52
70	VGSAICSeKAAVSKGIEVTSLSRSG	23	C6(MeSeCyS)	2282.131979	39.13

Table S5. Affinity and interaction sites between Keap1 (5WFV) and IPP-Se according to molecular docking simulations.

Peptides	Modifications	Residues	Binding energy (kcal/mol)

RCLVWFLK**C**R	C9(S-Se)	Arg483, Arg380, Ser508, Ser555	-6.5
FEA**C**LVMKHHL	C4(MeSeCys)	Arg483, Arg415, Asn414, Gln530, Tyr525, Gly527, Gln528	-6.3
FWGKK**C**VVTAGAW	C6(MeSeCys)	Arg380, Asn414, Gly480, Gly433	-6.2
KPDIL**M**LNSLIDMYG	M6(S-Se)	Arg483, Arg415, Ser508, Ser431, Tyr525, Tyr572, Asp389	-6.4
GASLI**C**IILLAIGKA	C6(S-Se)	Arg415, Ser555, Tyr525, Tyr572, Tyr334, Gly433,	-6.5
FSLWVIA**C**LDDWALL	C8(S-Se)	Arg483, Arg415, Gln530, Tyr525, Asn414, Tyr572,	-6.7
NPWIALRACAL**C**LPAI	C12(S-Se)	Val463, Val418, Val369, Val512, Val465, Gly371, Ile416	-6.6
SLLL**C**GLL**M**LIGVMLE	C5(MeSeCys);M9(S-Se)	Arg483, Asn382, Gln530, Tyr572, Asp529	-5.5
FAL**M**SLLGASIMPHNF	M4(S-Se)	Arg470, Val369, Val418, Val606, Gln563, Gly367, Ile559	-6.7
